# Economic and caregiver impact of Alzheimer’s disease across the disease spectrum: a cohort study

**DOI:** 10.1186/s13195-022-00969-x

**Published:** 2022-02-12

**Authors:** Virginie Dauphinot, Michele Potashman, Mihaela Levitchi-Benea, Ray Su, Ivana Rubino, Pierre Krolak-Salmon

**Affiliations:** 1 Clinical and Research Memory Centre of Lyon, Lyon Institute For Elderly, Lyon University Hospital, Lyon, France; 2 University of Lyon, Lyon, France; 3 Biogen, Cambridge, MA USA; 4 Neuroscience Research Centre of Lyon, Inserm 1048, CNRS, 5292 Lyon, France

**Keywords:** Costs of care, Economics, Alzheimer’s disease, Ccognitive status

## Abstract

**Background:**

Alzheimer’s disease (AD) substantially increases health-related costs. This study investigates direct medical costs and characterizes the caregiver burden across AD stages.

**Methods:**

This study analyzed data from the French Primary Health Insurance Fund claims database and reflected this public payer perspective. Outpatients (*N* = 1998) visiting a memory clinic at Lyon University Hospital in France between 2014 and 2019 were included. Real healthcare costs (ie, ambulatory medicine, paramedical care, pharmaceutical treatment, public and private hospital stays, and medical transportation) were collected for patients 1 year prior to the date of the first memory visit and 2 years following the first visit (reference year: 2019). Patients were grouped based on a clinical diagnosis of cognitively normal with a subjective cognitive complaint (SCC), all-cause mild cognitive impairment (MCI), or AD dementia. The severity of AD dementia was defined by the Mini-Mental State Examination score. Caregiver burden was measured using the mini Zarit Burden Interview. A generalized linear model was used for statistical analyses. Other patient nonmedical and indirect costs and caregiver costs were not included.

**Results:**

The study sample included patients with SCC (*n* = 640), MCI (*n* = 630), mild (*n* = 212), moderate (*n* = 256), or moderately severe/severe AD dementia (*n* = 260). One year after the first consultation, mean total costs were higher with progressive cognitive deficit, with little difference between dementia groups (SCC = €8028; MCI = €9758; mild AD dementia = €10,558; moderate AD dementia = €10,544; moderately severe/severe AD dementia = €10,345; *P* < .001). Public hospital stays comprised the majority of direct medical costs during the first semester following the visit (49.4% of the total costs), regardless of the severity of cognitive deficit. Caregiver burden increased with the severity of cognitive deficit (*P* < .0001).

**Conclusions:**

Direct medical costs and caregiver burden rose from SCC to AD dementia; in patients with AD dementia, the direct medical costs increased over the 2 years after the first consultation. These results, in conjunction with data from other care components, will be critical to elucidate the potential economic value of a therapeutic intervention that slows AD progression.

**Supplementary Information:**

The online version contains supplementary material available at 10.1186/s13195-022-00969-x.

## Background

Alzheimer’s disease (AD) substantially increases health-related costs in developed countries [1]. In 2015, dementia incurred a societal cost of €730 billion in the USA, rising from an estimated €452 billion in 2010. In Europe, the estimated cost of dementia was €105.2 billion in 2010 [2]. AD encompasses a continuum ranging from cognitively normal individuals with biomarker evidence of AD to individuals with severe AD dementia [3]. It is critical to evaluate the costs of resources used at every stage of the disease to more accurately estimate how future interventions will affect the economic costs of AD. While the immense socioeconomic burden of AD has been described, costs and burden related to specific stages along the AD continuum are not well characterized. For instance, most studies have estimated the average cost per patient without taking into account predementia stages [4–10]. Additionally, these costs were often presented with different amplitudes depending on population characteristics, study perspectives (payer or societal), cost components (direct, indirect, or informal), and time of evaluation, making comparisons difficult across studies [4–10]. Health expenditures associated with patient care are also limited, despite being an objective evaluation of costs related to patient care [10].

Here, we investigated medical and nonmedical direct costs, obtained from the local branch of the French Primary Health Insurance Fund (PHIF) in patients with a clinical diagnosis of cognitively normal with a subjective cognitive complaint (SCC), all-cause mild cognitive impairment (MCI), or mild, moderate, or moderately severe/severe AD dementia. We also investigate the caregiver burden associated with AD progression.

## Methods

### Study design and setting

This is an ancillary study from the MEMORA cohort, which aimed to investigate the determinants associated with functional change in individuals receiving routine care [11]. In the present study, the patients’ clinical data were matched 1 year before and 2 years after the first memory consultation with claims data from the local branch of the PHIF (Caisse Primaire d’Assurance Maladie du Rhône [CPAM]) database for patients from the MEMORA cohort who underwent a consultation at a memory clinic of the Lyon University Hospital in France between 2014 and 2019. This claims database included all the direct medical costs taken in charge by the national healthcare system, as well as medical transportation [12]. All patients had cognitive complaints, which were expressed by the patient or caregiver. Data matching led to a database including 4173 patients, which represented 61.2% of the initial MEMORA cohort in this period. The absence of data matching was due to wrong coding, misspelled names between the 2 databases, or because the patient depended on insurance other than the French PHIF, or, more specifically, the Rhône Primary Health Insurance Fund.

### Study population

Patients met the following inclusion criteria: living at home or in community-based housing, having coverage by the PHIF, having ≥ 1 year of follow-up, and having a clinical diagnosis at the first visit by SCC and a Mini-Mental State Examination (MMSE) score > 26, all-cause MCI and an MMSE score < 26, or AD dementia at mild, moderate, or moderately severe/severe stages defined by an MMSE score of 20 to 26, 15 to 19, or < 14, respectively. Exclusion criteria included patients under legal protection. Information regarding the collection of individual data, with the aim of performing research on routine care, was provided to the patients. This ancillary research of the MEMORA study was considered noninterventional by the local ethics committee CPP Lyon Sud-Est IV (Comité de Protection des Personnes [Committee for the Protection of People]). No signed consent was required for participation, but patients may refuse the use of their data for research purposes. Authorization for handling personal data was granted by the representative of the French Data Protection Authority, the Commission Nationale de l’Informatique et Libertés. All procedures are in compliance with the Declaration of Helsinki. The MEMORA cohort also obtained agreements from the regional ethics committee, Comité de Protection des Personnes Sud Est III and the French Data Protection Authority (ClinicalTrials.gov code: NCT02302482).

### Primary outcome

Primary outcomes included source of healthcare costs, components of claims data, valorization of the costs, and caregiver impact. First, healthcare costs, reflecting a healthcare payer perspective, were assessed for each care, act, and treatment that occurred for the insurance recipient and was reimbursed through the local branch of the PHIF. Claims data included all medical direct costs and the one nonmedical direct cost, medical transportation, which was supported by the local PHIF; PHIF is the equivalent of the national claims database described elsewhere [13].

Second, components of claim data included (1) ambulatory medicine (e.g., consultations and care provided by general practitioners, neurologists, psychiatrists, and geriatricians) and other ambulatory care (e.g., surgical procedures in private practice, ophthalmologic and hearing devices, dental care, biological analyses, radiology examinations, immunization, home dialysis, at-home hospitalizations, and health cures); (2) paramedical care from nurses, physiotherapists, and others, such as speech therapists or orthoptists; (3) pharmaceutical treatment from retail pharmacies for AD medication, psychotics/hypnotics, and other pharmaceutical treatment using the Anatomical Therapeutic Chemical classification of the World Health Organization; (4) costs related to hospital stays in geriatric wards, psychiatric wards, internal medicine wards, and surgery wards and all other costs related to hospital stays; and (5) private hospital stays. Medical transportation is the only nonmedical direct cost covered by the PHIF that was considered in this study.

Third, valorization of costs was presented as constant costs adjusted for the value of the euro in 2019 according to Institut Nationale de la Statistique et des Etudes Economiques (French national institute for statistical and economic studies; https://www.insee.fr/fr/information/2417794). Costs per patient were expressed as the sum of the costs per semester and per year (for the total costs), both overall and according to the item of interest, within the time frame of 1 year prior to the date of the first memory visit and 2 years following the first visit. Total costs for patients who died were considered in the corresponding semester. Costs related to a nursing home were not included for all groups. Notably, the PHIF applies a specific reimbursement level, which is similar nationally, for each care, act, and treatment. However, reimbursement rates may vary according to several criteria; details are publicly available at https://www.service-public.fr/particuliers/vosdroits/N418. The prices and rates taken in charge by the PHIF are listed in Supplementary Table 1. The average costs paid by the patient and the estimated costs reimbursed by the PHIF are also presented.

Fourth, caregiver burden was assessed using the short version of the Zarit Burden Interview (sZBI) during a face-to-face interview between the caregiver and nurses during the memory consultation [14]. A higher sZBI score indicates a higher burden (range of 0 to 7).

### Collection of data

Baseline characteristics were collected at the initial visit. Clinical data were collected at the memory consultation by trained nurses and administrative staff and recorded in an electronic case report form. Cognitive performance was assessed using the MMSE. The total score ranges from 0 to 30, and a lower score indicates increased cognitive impairment [15]. Functional abilities were evaluated using the Lawton Instrumental Abilities of Daily Living (IADL) scale; the summary score ranges from 0 to 8, with 8 indicating autonomy [16]. The behavioral and psychological symptoms of dementia were assessed using the Neuropsychiatric Inventory (NPI); the total NPI score ranges from 0 to 144, and 144 indicates a severe behavioral disorder [17]. Clinical diagnosis was determined by a neurologist, geriatrician, or psychiatrist based on clinical assessments and by using the MMSE score. Patients were diagnosed with SCC when they performed normally on the neuropsychological assessment but the patient expressed a cognitive complaint or the caregiver reported one for the patient; in addition, patients had to have an MMSE score ≥ 26 at the time of diagnosis. A diagnosis of MCI or AD dementia was established using the 2011 National Institute on Aging-Alzheimer’s Association criteria and *the Diagnostic and Statistical Manual of Mental Disorders, Fifth Edition* [18–20]. In addition, patients with MCI or AD dementia had to have an MMSE score < 26 at the time of diagnosis. Dementia severity was assessed using the MMSE score: 20 to 26 for mild AD dementia, 15 to 19 for moderate AD dementia, and ≤ 14 for moderately severe/severe AD dementia. These thresholds were similar to those used in previous studies [21, 22].

The dates of nursing home admission and death and the treatments for comorbidities (e.g., arterial hypertension, diabetes mellitus, hypercholesterolemia, depression, and anxiety) were collected from the PHIF database. During the study, a patient was considered to have a comorbidity if they received reimbursement of treatment for one.

### Statistical analysis

Patient demographics were presented as mean ± standard deviation (SD) or frequency (percentage). Between-group comparisons were performed using analysis of variance or Pearson chi-squared test. Paired-wise comparisons were made using post hoc Bonferroni and Sheffé tests. The occurrence of nursing home admission and death was described according to the semester and during the study period (2014–2019).

Costs per patient were described in euros per semester and per year and according to the diagnosis. Total costs were classified as medical or nonmedical and categorized according to the different origins of the costs, using means (SD, SE, or 95% CI) and medians (IQR). Total costs were also described by age (≥ 80 years vs. < 80 years), gender, educational level (≥ 12 years vs. < 12 years), and treatment for comorbidities.

Total costs were compared between groups by semester using generalized linear models (GLM) with gamma distribution and log link [23]. As the nonmedical direct costs of medical transportation did not represent a large amount, these costs were added to the medical direct costs to calculate a total cost per patient. The sZBI scores were compared between groups using GLM. P values unadjusted and adjusted for age, gender, and education level were reported. Analyses were performed using Statistical Package for the Social Sciences (SPSS) version 19.0 for Windows (SPSS Inc., Chicago, Illinois, USA).

### Sensitivity analyses

Due to large differences between mean and median costs, patients with costs ≥ 3 SDs from the mean were identified as outliers. Outlier patient characteristics and resource utilization, as well as the implications of removing outliers from analyses, were assessed. A Student *t*-test or the Pearson chi-squared test was used to assess patient characteristics with 3 SDs above or below average total costs. The association between diagnosis and total costs was tested when excluding outliers using GLM with log link and gamma distribution.

## Results

### Patient demographics

In patients (*N* = 1998) included in the study, 640 were cognitively normal with SCC; 630 patients had MCI of unspecified etiology; and 212, 256, and 260 patients had mild, moderate, or moderately severe/severe AD dementia, respectively (Table 1, Supplementary Fig. 1). Most patients (52.9%) were aged ≥ 80 years or over, except in the SCC group (33.3%); 63% were female. There were fewer patients in the moderate and severe AD dementia groups with an education level > 12 years compared with other groups (*P* < .001). The mean MMSE decreased significantly with the severity of cognitive deficit (*P* < .001). Similarly, the IADL score decreased with cognitive deficit severity (*P* < .001). However, no significant difference in IADL scores occurred between the mild AD dementia (mean IADL = 4 ± 2) and moderate AD dementia groups (mean IADL = 3.6 ± 2); a post hoc test result was 0.16. Further, the proportion of patients treated for depression (*P* < .001) was significantly higher in the AD dementia group compared with other groups.

**Table 1:** Description of the study population

	Total	SCC	MCI	Mild AD dementia	Moderate AD dementia	Moderately severe/severe AD dementia	***P*** value*
	(***N*** = 1998)	(***n*** = 640)	(***n*** = 630)	(***n*** = 212)	(***n*** = 256)	(***n*** = 260)	
Age, mean (SD)	78.62 (8.61)	74.58 (9.98)	79.02 (7.62)	81.69 (6.63)	82.37 (5.97)	81.41 (6.63)	< .001
Sex, *n* (%)							
Female	1259 (63.00)	369 (57.66)	388 (61.59)	131 (61.79)	185 (72.27)	186 (71.54)	< .001
Male	739 (37.00)	271 (42.34)	242 (38.41)	81 (38.21)	71 (27.73)	74 (28.46)	
Education level, *n* (%)							
≥ 12 years	456 (22.4)	319 (49.84)	152 (24.13)	66 (31.13)	44 (17.19)	29 (11.15)	< .001
< 12 years	1306 (64.1)	280 (43.75)	447 (70.95)	146 (68.87)	212 (82.81)	230 (88.46)	
Unknown	276 (13.5)	41 (6.41)	31 (4.92)	0	0	1 (0.38)	
MMSE score (out of 30), mean (SD) (*n* = 1962)	22.08 (6.14)	28.02 (1.32)	23.28 (1.95)	22.36 (1.90)	17.12 (1.45)	10.07 (3.43)	< .001
IADL (out of 8), mean (SD)	5.06 (2.40)	6.57 (1.79)	5.49 (2.09)	4.01 (2.04)	3.62 (1.97)	2.54 (1.83)	< .001
NPI (out of 144), mean (SD) (*n* = 1396)	18.49 (16.11)	16.41 (15.14)	15.75 (15.17)	19.16 (16.69)	21.01 (15.74)	23.25 (17.45)	< .001
Received treatment for comorbidities, *n* (%)							
Hypertension	1294 (64.76)	368 (57.50)	449 (71.27)	147 (69.34)	153 (59.77)	177 (69.08)	< .0001
Diabetes mellitus	360 (18)	110 (17.19)	112 (17.78)	49 (23.11)	36 (14.06)	53 (20.38)	.10
Hypercholesterolemia	731 (36.59)	212 (33.12)	273 (43.33)	78 (36.79)	82 (32.03)	86 (33.08)	< .0001
Depression	902 (45.15)	256 (40)	270 (42.86)	108 (50.94)	122 (47.66)	146 (56.15)	< .0001
Anxiety	650 (32.53)	188 (29.38)	210 (33.33)	63 (29.72)	85 (33.20)	104 (40)	.033

*AD* Alzheimer’s disease, *IADL* Lawton Instrumental Abilities of Daily Living, *MCI* mild cognitive impairment, *MMSE* Mini-Mental State Examination, *NPI* Neuropsychiatric Inventory, *SCC* subjective cognitive complaint, *SD* standard deviation

*ANOVA or chi^2^ of Pearson test

### Costs according to the severity of cognitive deficit

When comparing the total costs by diagnosis, the mean total costs 1 year after the first consultation tended to increase with the severity of cognitive deficit (SCC = €8028; MCI = €9758; mild AD dementia = €10,558; moderate AD dementia = €10,544; moderately severe/severe AD dementia = €10,345; *P* < .001) (Table 2). The analysis per semester showed similar trends. Mean total costs in the first semester were numerically higher for the MCI and AD dementia groups compared with the SCC group (SCC = €4578; MCI = €4876; mild AD dementia = €5282; moderate AD dementia = €5443; severe AD dementia = €4969; Supplementary Table 2).

**Table 2:** Costs per year according to the severity of the diagnosis

			1 year before	First year after	Second year after
All patients	*N*		1928	1998	1600
	Missing, *n*		70	0	398
	Total costs, €	Mean	7408	9466	8870
		SD	14,941	17,664	15,759
		SE	340	395	394
		Median	2054	3444	3249
		IQR	2705	4780	4843
	Direct medical costs, €	Mean	7101	9103	8590
		SD	14,081	17,097	15,448
		SE	321	383	386
		Median	2014	3313	3088
		IQR	2613	4674	4662
	Direct nonmedical costs (transportation), €	Mean	307	363	280
		SD	4132	2758	2212
		SE	180	102	92
		Median	166	288	262
		IQR	254	399	325
SCC	n		619	640	513
	Missing, *n*		21	0	127
	Total costs, €	Mean	7104	8028	6196
		SD	14,964	15,016	11,917
		SE	601	594	526
		Median	1798	2576	1811
		IQR	2285	3289	2915
	Direct medical costs, €	Mean	6604	7581	5885
		SD	13,234	14,011	11,365
		SE	532	554	502
		Median	1764	2532	1802
		IQR	2128	3223	2747
	Direct nonmedical costs (transportation), €	Mean	500	447	310
		SD	5941	3678	3150
		SE	480	261	268
		Median	153	266	233
		IQR	227	433	240
MCI	*n*		610	630	511
	Missing, *n*		20	0	119
	Total costs, €	Mean	7899	9758	8452
		SD	15,626	18,297	14,291
		SE	633	729	632
		Median	2160	3486	3286
		IQR	2588	4672	4639
	Direct medical costs, €	Mean	7596	9361	8130
		SD	14,946	17,665	13,849
		SE	605	704	613
		Median	2130	3329	3073
		IQR	2563	4591	4180
	Direct nonmedical costs (transportation), €	Mean	303	397	323
		SD	4143	3088	2553
		SE	310	194	183
		Median	185	357	309
		IQR	310	452	447
Mild AD dementia	*n*		204	212	173
	Missing, *n*		8	0	39
	Total costs, €	Mean	8022	10,558	13,717
		SD	18,658	18,583	24,093
		SE	1306	1276	1832
		Median	2370	5136	5512
		IQR	2861	5275	4695
	Direct medical costs, €	Mean	7889	10,244	13,462
		SD	18,426	18,317	23,946
		SE	1290	1258	1821
		Median	2358	4765	5512
		IQR	2867	5434	4835
	Direct nonmedical costs (transportation), €	Mean	133	314	254
		SD	755	1088	909
		SE	101	115	106
		Median	187	294	283
		IQR	324	462	274
Moderate AD dementia	*n*		242	256	208
	Missing, *n*		14	0	48
	Total costs, €	Mean	6081	10544	9783
		SD	10,362	18,688	14,155
		SE	666	1168	981
		Median	2047	4384	4145
		IQR	3682	5672	6257
	Direct medical costs, €	Mean	5977	10,256	9589
		SD	10,167	18,377	13,993
		SE	654	1149	970
		Median	2018	4292	4095
		IQR	3682	5856	6446
	Direct nonmedical costs (transportation), €	Mean	104	288	193
		SD	811	1349	847
		SE	98	140	98
		Median	102	263	280
		IQR	103	325	297
Moderately severe/severe AD dementia	*n*		253	260	195
	Missing, *n*		7	0	65
	Total costs, €	Mean	7740	10,345	11,728
		SD	13,483	20,077	18,903
		SE	848	1245	1354
		Median	2212	4130	4993
		IQR	4613	6323	8169
	Direct medical costs, €	Mean	7563	10,161	11,525
		SD	13,090	19,914	18,810
		SE	823	1235	1347
		Median	2207	4125	4448
		IQR	4603	6222	7857
	Direct nonmedical costs (transportation), €	Mean	178	184	203
		SD	1975	1042	615
		SE	238	109	64
		Median	212	229	213
		IQR	230	236	217
*P* value*			0.096	0.004	< .0001
*P* value^†^			0.487	0.225	< .0001

*AD* Alzheimer’s disease, *GLM* generalized linear model, *IQR* interquartile range, *MCI* mild cognitive impairment, *SCC* subjective cognitive complaint, *SD* standard deviation, *SE* standard error

*GLM to compare mean total costs between the different diagnoses

^†^GLM adjusted for age, sex, and education level

### Analysis of costs details

**Fig. 1:**
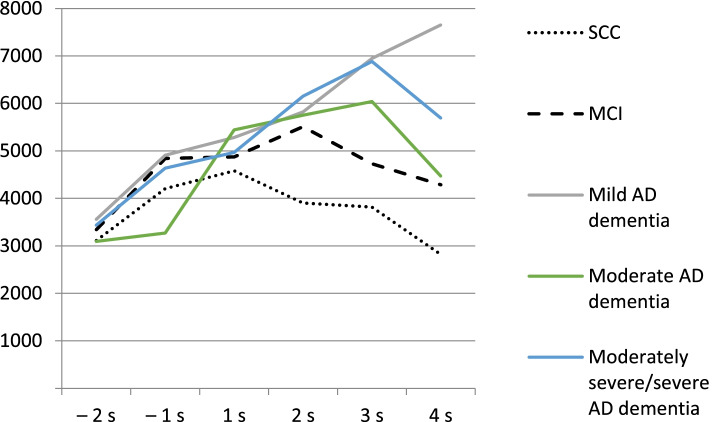
Total costs in euros per semester across the disease stages. AD, Alzheimer’s disease; MCI, mild cognitive impairment; SCC, subjective cognitive complaint. −2s: second semester before the memory visit; 4s: fourth semester after the memory visit

Regardless of the patient diagnosis, costs were highest 1 year after the first visit (Fig. 1; Supplementary Table 2). In the SCC and MCI groups, mean annual total costs increased in the first year after diagnosis but decreased in the second year; costs also decreased in the moderate AD dementia group, but the decrease was more slight (Table 2). For mild AD and moderately severe/severe AD dementia groups, mean annual total costs increased for 2 years after diagnosis.

Examining cost details revealed that most of the total direct medical costs were explained by public hospital stays, followed by paramedical care, for all groups (Supplementary Table 3). Public hospital stays represented an average of 49.4% of the total costs during the first semester after the first visit, and paramedical care represented an average of 20.4% of the total costs. The total ambulatory costs were lower, whereas the total paramedical costs were higher in AD dementia groups compared with SCC and MCI groups. General practitioner visits represented an average of 10.1% of the total ambulatory costs for the first semester after the first visit. The costs associated with paramedical care were mostly due to care by nurse (76% of the total paramedical costs for the first semester after the first visit). AD medication costs increased with diagnosis severity but represented a modest proportion (10.6% for the first semester after the first visit) of the costs associated with pharmaceutical treatment compared with all other medications combined. Stays in public geriatric and psychiatric wards accounted for 35.6% of the total costs of stays in public hospitals. Costs are described in more detail in Supplementary Table 4.

### Costs according to patient demographics

Total costs by age, sex, education level, and treatment for comorbidities are presented in Supplementary Table 5. Mean total costs were higher for patients > 80 years and for patients with less education. Mean total costs were higher for men than women the year before diagnosis and the first semester after. Total costs were numerically higher for patients treated for comorbidities of interest.

### Caregiver burden according to the severity of cognitive deficit

**Fig. 2:**
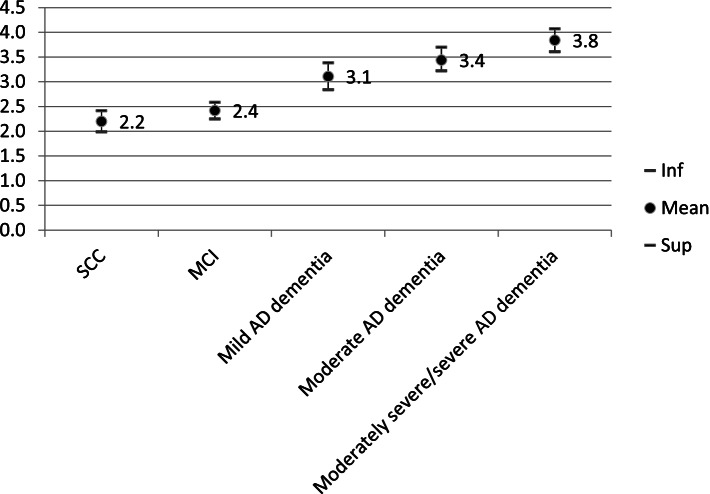
Caregiver burden across the disease stages. * sZBI scores range from 0 to 7, with a higher number corresponding to increased caregiver burden. AD, Alzheimer’s disease; MCI, mild cognitive impairment; Inf, limit inferior of the 95% confidence interval, SCC, subjective cognitive complaint; Sup, limit superior of the 95% confidence interval. (*N* = 1367; *P* < .0001)

The association between caregiver burden and disease severity was assessed in 1367 patients. Caregiver burden increased with disease severity independent of age and sex (mean sZBI scores: SCC = 2.2 ± 1.9; MCI = 2.4 ± 1.8; mild AD dementia = 3.1 ± 1.8; moderate AD dementia = 3.4 ± 1.8; moderately severe/severe AD dementia = 3.8 ± 1.8; *P* < .001) (Fig. 2).

### Sensitivity analysis

When outlier patients with costs ≥ 3 SDs above-average total costs were excluded, mean values better reflected the median. In the first year after the initial visit, the SCC group had a mean total cost of €5989, with a median cost of €1709 (Table 3). Furthermore, in the mild AD dementia group, the mean cost was €7336, and the median was €4670. The trend for costs to increase with disease severity (in patients with SCC, MCI, or AD dementia) was preserved in the sensitivity analysis (Table 3; Supplementary Table 6). In the AD dementia groups, the average total costs were numerically higher in the moderately severe/severe AD dementia group (€7834) compared with the mild (€7336) and moderate AD (€7315) groups. The evolution of the average total costs over time remained similar in the sensitivity analysis, except in the moderate AD dementia group, for which the average total costs increased both years after the initial visit, similar to the mild and moderately severe/severe AD dementia groups.

**Table 3:** Mean total cost/year after exclusion of costs ≥ 3 SD of the average total costs

Costs, €		1 year before	1 year after	2 years after
SCC	Mean	4460	5989	4749
	SE	314	353	332
	Median	1709	2451	1761
	SD	7637	8803	7424
	IQR	1991	3017	2754
MCI	Mean	5514	7272	6629
	SE	354	394	400
	Median	1979	3339	3074
	SD	8580	9728	8913
	IQR	2354	4277	4050
Mild AD dementia	Mean	6021	7336	7771
	SE	623	563	761
	Median	2323	4670	4796
	SD	8815	8045	9591
	IQR	2826	4542	4190
Moderate AD dementia	Mean	5320	7315	7741
	SE	499	561	681
	Median	1994	4085	3983
	SD	7715	8776	9631
	IQR	3464	5152	5790
Moderately severe/severe AD dementia	Mean	6148	7834	8303
	SE	529	638	724
	Median	2160	3909	4453
	SD	8316	10141	9880
	IQR	3891	5907	7425
*P* value*		0.002	0.006	<0.0001
*P* value^†^		0.065	0.409	<0.0001

*AD* Alzheimer’s disease, *GLM* generalized linear model, *IQR* interquartile range, *MCI* mild cognitive impairment, *SCC* subjective cognitive complaint, *SD* standard deviation, *SE* standard error

*GLM to compare mean total costs between the different diagnoses

^†^GLM adjusted for age, sex, and education level

When comparing the patient characteristics of outliers (*n* = 51) with the rest of the population (*n* = 1947) in the first semester, outliers were more frequently treated for anxiety (49% vs. 32.1%; *P* = .011) and diabetes mellitus (31.4% vs. 17.7%; *P* = .012) (Supplementary Table 7), and the IADL score was lower in the outlier group than in the rest of the population (IADL, 3.8 vs. 5.1; *P* < .0001).

## Discussion

In this study, we describe direct medical costs and medical transportation costs according to the severity of cognitive impairment. Direct medical and nonmedical (ie, medical transportation) costs and caregiver burden significantly increased with greater progression of cognitive deficit in the SCC, MCI, and AD dementia groups. Costs increased with disease severity when assessed both before and after the initial visit. These results build on previous findings suggesting that greater cognitive impairment results in higher direct medical costs and higher caregiver impact in various settings [7, 9, 21, 22, 24–33].

Although there was a trend of increased cost with greater cognitive decline, medical costs were similar the first year after diagnosis among the different cognitive stages in AD dementia. This finding is consistent with those of previous studies that did not highlight significant differences among the severity stages in AD dementia [5, 6, 34, 35] (Supplementary Table 8), while others studies have shown that the healthcare costs increased among different cognitive stages of AD dementia [21, 27, 36, 37]. In our study, similar costs between the different cognitive stages in AD dementia may be explained by the fact that patients with higher healthcare costs are less represented in the more severe stages of AD dementia due to nursing home admission or death. In our study, the proportions of nursing home admissions and deaths were higher in the moderately severe/severe stage of AD dementia than in the less severe stages (Supplementary Table 9), and the costs related to nursing homes are not covered by the PHIF. In a post hoc analysis, where the patients who were admitted to a nursing home or died were excluded, the average direct medical cost was €7178 in the mild AD group, €7164 in the moderate AD group, and €8264 in the moderately severe/severe AD group.

Another possible explanation is that the costs may shift from the medical sector covered by the PHIF to other social care sectors. While this study presented the direct medical costs covered by the local PHIF, previous studies have shown increased patient social care and caregiver informal care costs with the cognitive severity groups in AD dementia, while the patient healthcare costs were similar or decreased between the groups [5, 6, 34]. Finally, when outlier patients with costs ≥ 3 SDs above-average total costs were removed from the sensitivity analysis, we found that medical costs were numerically higher in the more severe AD dementia group, which may mean that extreme costs are related to medical conditions other than cognitive decline.

Higher healthcare costs were observed in this study compared with those in the French GERAS cohort, which analyzed data from patients at memory clinics with mild, moderate, or moderately severe/severe AD dementia. These higher costs in the MEMORA cohort were observed even after accounting for differences in the years that the costs were reported [37]. In the GERAS study, healthcare costs over 18 months were €5129 for mild AD dementia, €7106 for moderate AD dementia, and €8118 for moderately severe/severe AD dementia [37]. In contrast, costs 1 year after the first visit to a memory clinic reported in our study were €10,558€ for mild AD dementia (€7336 in the sensitivity analysis), €10,544 for moderate AD dementia (€7315 in the sensitivity analysis), and €10,345 for moderately severe/severe AD dementia (€7834 in the sensitivity analysis). The MMSE was used to define the severity of AD dementia in both studies, and the mean age and mean MMSE scores at baseline were comparable for each level of disease severity, suggesting that these factors did not impact differences in costs. Given this, it is likely that the cost differences between the present study and GERAS cohorts differed because GERAS captured healthcare resources using the Resource Utilization in Dementia instrument, which is patient and caregiver reported, rather than analyzing data extracted from the PHIF claims database. Also, the present study included all direct medical costs of patients visiting a memory clinic, even those related to medical conditions other than AD dementia.

In our study, the majority of direct medical costs were incurred during public hospital stays, which is consistent across studies in Europe and the US [10, 21].

Regardless of the disease stage, costs increased the year after the first consultation. Two years after the first consultation, the evolution of costs varied according to the patient group: The average total cost decreased in patients with SCC and MCI and increased in the AD dementia groups. An explanation for this result may be that another medical condition caused the patient to enter into the healthcare system initially; thus, the bulk of the costs in the first year could be associated with another medical condition unrelated to cognitive impairment. Alternatively, cognitive decline may have been the rationale to seek a memory clinic consultation, which may have led to increased care services to confirm a diagnosis and to create an individualized care or management plan [38].

Total costs were higher for older patients and patients treated for comorbidities (i.e., arterial hypertension, diabetes mellitus, hypercholesterolemia, depression, and anxiety), which is consistent with previous studies [39]. Additionally, the observed increase in disease burden with AD progression, from mild to moderately severe/severe AD dementia, is also consistent with the previous literature [40]. Importantly, this study extends previous work by addressing the burden of disease at earlier stages of cognitive decline (e.g., SCC and MCI). Results showed that the burden of disease is comparable across these early disease stages.

As typical in costs analyses, there were some patients who accumulated particularly high costs, resulting in discrepancies between means and medians. Skewed data was accounted for using gamma function in the models, and a sensitivity analysis was performed with statistically significant, high-costs outliers removed from the sample. Results of the sensitivity analysis showed that patients with extremely high costs were generally in poorer health and had a higher incidence of anxiety, which is consistent with previous studies [41, 42]. As such, these factors help explain the elevated costs associated with these patients.

In this study, direct medical and nondirect transportation costs were assessed using information from the PHIF claims database. To our knowledge, this is the first study to describe direct medical costs and medical transportation costs according to the severity of cognitive impairment in France from the perspective of the public payer and to report the costs before and after an initial memory clinic visit. Approximately 90% of the French population is covered by the PHIF; therefore, using this database permitted a reliable representation of the health spending from the public payer [13].

### Limitations

Besides transportation, other nonmedical direct costs, such as home support and indirect costs, were not included in cost analyses because these costs are not covered by the PHIF. As such, nonmedical costs, like informal care time, which may represent 50% to 80% of the overall costs associated with AD depending on the study [35, 37], should be taken into consideration in future studies evaluating the total economic cost of AD. Moreover, all costs covered by the PHIF were considered in this study, including those that are not specifically related to AD. This may have led to an overestimation of the direct medical costs; nevertheless, this overestimation may be present for all groups compared in this study. Also of note, the healthcare coverage of the PHIF is specific to the French national system. National health policies differ between countries and can change over time; in France, for example, reimbursement for drugs used to treat AD (donepezil, galantamine, memantine, and rivastigmine) ended in 2018. The health care that is not reimbursed by the PHIF may be reimbursed by private insurance or paid by the patients or their families.

The neurocognitive diagnosis was determined at the first visit; thus, it is important to consider that the evolution of costs over time may have been accompanied by the decline of patients’ health statuses, particularly cognitive and functional impairment in the AD dementia groups. It is less certain for patients in the SCC and the MCI groups, whose disease may not progress to a more advanced stage. Although the data did not allow us to show patients’ health status decline, the increase in nursing home admissions and deaths over time supports this hypothesis (Supplementary Table 9).

AD was diagnosed based on clinical and neuropsychological evaluations and was not confirmed by biomarkers. Future studies should include biomarker assessment during diagnosis. The present study is limited to findings from an older French population at 1 memory clinic; however, the patients’ characteristics were comparable to those of the multicentric GERAS cohort in France [37]. Nevertheless, it will be important to conduct cost analyses across other geographical and social policy contexts.

## Conclusions

The economic toll of AD is debilitating and continues to grow. These results show that direct medical costs and caregiver burden rise with the severity of cognitive impairment from SCC to AD dementia; they were similar in the AD dementia groups that were determined by the MMSE. In AD dementia, the direct medical costs increased 2 years after the first consultation in a memory clinic. These results, in conjunction with data from other care components, will be critical in revealing the potential economic value of a therapeutic intervention that halts or slows the progression of AD.

## Supplementary Information

**Additional file 1: Table S1**.

**Additional file 2: Figure S1.** Flowchart.

**Additional file 3: Table S2.** Cost by Semester.

**Additional file 4: Table S3.** Cost by Domain 1.

**Additional file 5: Table S4.** Cost by Domain 2.

**Additional file 6: Table S5.** Cost by Characteristics.

**Additional file 7: Table S6.** Sensitivity.

**Additional file 8: Table S7.** Characteristics of patients.

**Additional file 9: Table S8.** Other Studies.

**Additional file 10: Table S9.** Events.

## Data Availability

The data sets analyzed in the present study are not publicly available due to regulations; the ethics board and the French Data Protection Authority, as well as the patients, were informed that their data would be analyzed only by the French Lyon University Hospital, and agreements obtained to perform the study (the contract between the local branch of the PHIF and the Lyon University Hospital) state that the claims data will not be transmitted outside the Lyon University Hospital. Collaborations to further explore these data are welcomed on request to the corresponding or last author. The data of the MEMORA study and this ancillary study are the property of the French Lyon University Hospital, France.

## Abbreviations

AD: Alzheimer’s disease
CPAM: Caisse Primaire d’Assurance Maladie du Rhône
GLM: Generalized linear models
IADL: Lawton Instrumental Abilities of Daily Living
IQR: Interquartile range
MCI: Mild cognitive impairment
MMSE: Mini-Mental State Examination
NPI: Neuropsychiatric Inventory
PHIF: Primary Health Insurance Fund
SCC: Subjective cognitive complaint
SD: Standard deviation
SE: Standard error
SPSS: Statistical Package for the Social Sciences
sZBI: Short version of the Zarit Burden Interview
