# Supplementary material for: Economic and caregiver impact of Alzheimer’s disease across the disease spectrum: a cohort study
Source: Alzheimers Res Ther. 2022 Feb 12;14:34. doi: 10.1186/s13195-022-00969-x (PMC8841058; doi:10.1186/s13195-022-00969-x)
Supplement: Supplementary file 1 — Additional file 1: Table S1. [file 13195_2022_969_MOESM1_ESM.doc]

**SUPPLEMENTARY MATERIAL**

**Supplementary Table 1: Price per health care resource in France, in the perspective of the French Primary Health Insurance Fund (PHIF)**

| **Domain of cares** | **Unit** | **Price per unit** | **Rate taken in charge by the PHIF** | **Mean cost per unit paid by the patient ± SD^a^** | **Mean cost per unit in the study reimbursed by the PHIF ± SD^b^** | **Source (access in August 2021)** |
| --- | --- | --- | --- | --- | --- | --- |
| **Ambulatory medicine** |  |  |  |  |  |  |
| **General practitioner (sector 1)** | 1 medical consultation | Sector 1: 23 € until 04/30/2017  25 € after 05/01/2017 | Sector 1: 70% or 100% | 29.1 € ± 7.9  (No distinction between sectors was possible in the study) | 23.3 € ± 6.9  (No distinction between sectors was possible in the study) | https://www.ameli.fr/rhone/assure/remboursements/rembourse/consultations/modifications-tarifs-consultations-mai-2017 A visit to a doctor in sector 1 has a fixed price without extra fees (94% of the GP are in sector 1). In sector 2, the price can be set freely. Price may vary depending on the situations e.g. different sectors, complex consultations, patients with illness of long duration (ALD) or with modest ressources and complementary universal health coverage (CSS/CMU-C), for which 100% of the costs are covered. |
| **Neurologist** | 1 medical consultation | Sector 1: 46.7 € | Sector 1: 70% or 100% | 64.5 € ± 15.1 | 48.8 € ± 13.6 | https://www.ameli.fr/rhone/assure/remboursements/rembourse/consultations/metropole |
| **Psychiatrist** | 1 medical consultation | Sector 1: 46.7 € | Sector 1: 70% or 100% | 52 € ± 16.7 | 35.9 € ± 8.5 |
| **Geriatrician** | 1 medical consultation | Sector 1: 23 € until 04/30/2017  25 € after 05/01/2017 | Sector 1: 70% or 100% | 32.5 € ± 11.3 | 27.1 € ± 10.7 |
| **Medical transportation** | 1 transportation | Vary according to the type of transportation and conditions | 65% or 100% | 67.1 € ± 44.4 | 63.2 € ± 40 | https://www.ameli.fr/rhone/assure/remboursements/rembourse/frais-transport/frais-transport |
| **Others (surgical procedures in private practice, ophthalmologic and hearing devices, dental care, biological analyses, radiology examinations, immunization, home dialysis, at-home hospitalizations, and health cures)** | 1 examination or 1 act or procedure, or 1 day |  | Vary | 46.3 € ± 88.9 | 32.6 € ± 42.9 | https://www.ameli.fr/rhone/assure/remboursements/rembourse/tableau-recapitulatif-taux-remboursement/tableau-recapitulatif-taux-remboursement |
|  |  |  |  |  |  |  |
| **Paramedical care** |  |  |  |  |  |  |
| **Nurse** | 1 visit | 16.13 € | 60% or 100% | 13.8 € ± 9.4 | 12.7 € ± 9.6 | https://www.ameli.fr/rhone/assure/remboursements/rembourse/tableau-recapitulatif-taux-remboursement/tableau-recapitulatif-taux-remboursement |
| **Physiotherapist** | 1 visit | 16.13 € | 60% or 100% | 21.5 € ± 3.9 | 18.7 € ± 5.7 |
| **Others (speech therapist, orthoptist…)** | 1 visit | 16.13 € | 60% or 100% | 40 € ± 14.6 | 36.7 € ± 15.2 |
|  |  |  |  |  |  |  |
| **Pharmaceutical treatment** |  |  | 30% to 100% |  |  |  |
| **Drugs used in hypertension (ATC codes: C02, C03, C07, C08, C09)** | 1 pack |  |  | 9.3 € ± 8.3 | 8 € ± 7.3 | https://www.whocc.no/atc_ddd_index/ https://www.ameli.fr/rhone/assure/remboursements/rembourse/tableau-recapitulatif-taux-remboursement/tableau-recapitulatif-taux-remboursement. The reimbursement of the drugs used in dementia by the PHIF was stopped in August 2018. Ref: Krolak-Salmon P, Dubois B, Sellal F, Delabrousse-Mayoux JP, Vandel P, Amieva H, Jeandel C, Andrieu S, Perret-Liaudet A. France Will No More Reimburse Available Symptomatic Drugs Against Alzheimer's Disease.J Alzheimers Dis. 2018;66(2):425-427. doi: 10.3233/JAD-180843. |
| **Drugs used in diabetes (ATC code: A10)** | 1 pack |  |  | 20.7 € ± 18.7 | 20 € ± 18.5 |
| **Drugs used in hypercholesterolemia (ATC code: C10)** | 1 pack |  |  | 17.2 € ± 17.6 | 15.5 € ± 16.4 |
| **Antidepressants (ATC codes: N06A, N06CA)** | 1 pack |  |  | 6.8 € ± 5.1 | 5.9 € ± 4.6 |
| **Anxiolytics (ATC code: N05B)** | 1 pack |  |  | 1.7 € ± 0.8 | 1.4 € ± 0.7 |
| **Antipsycholeptics (ATC code: N05A)** | 1 pack |  |  | 12.9 € ± 14.4 | 11.9 € ± 14.1 |
| **Hypnotics and sedatives (ATC code: N05C)** | 1 pack |  |  | 2.1 € ± 0.5 | 1.3 € ± 0.9 |
| **Drugs used in dementia (ATC code: N06DA, N06DX01) (estimated until 2018)** | 1 pack |  |  | 42.7 € ± 6.2 | 39.3 € ± 5.2 |
| **Others** | 1 pack |  |  | 11.7 € ± 30.5 | 8 € ± 7.3 |
|  |  |  |  |  |  |  |
| **Hospital stays in public hospital** | Per day/night |  |  | Total cost / number of days in hospital stay | Total cost / number of days of hospital stay | https://www.ameli.fr/rhone/assure/remboursements/rembourse/tableau-recapitulatif-taux-remboursement/tableau-recapitulatif-taux-remboursement |
| **Geriatric wards** |  | 929 €* | 80% or 100% | Not available | 1205.5 € ± 432.3 | *Based on the statistics of the ATIH in 2016 https://www.scansante.fr/applications/couts-UO |
| **Psychiatric wards** |  | 347 €* | 80% or 100% | Not available | 602.9 € ± 307.4 | *https://www.scansante.fr/applications/couts-UO |
| **Internal medicine wards** |  | 1204 €* | 80% or 100% | Not available | 1418.0 € ± 193.5 | *https://www.scansante.fr/applications/couts-UO |
| **Surgical wards** |  | 1370 €* | 80% or 100% | Not available | 1761.5 € ± 568.3 | *https://www.scansante.fr/applications/couts-UO |
| **Others** |  | 1096 €† | 80% or 100% | Not available | 1129.3 € ± 583.67 | †cost for a day of hospitilization, whatever the speciality / https://www.scansante.fr/applications/couts-UO |
|  |  |  |  |  |  |  |
| **Hospital stays in private hospital** | Per day/night |  | 80% or 100% | Not available | 540.4 € ± 558.3 | https://www.ameli.fr/rhone/assure/remboursements/rembourse/tableau-recapitulatif-taux-remboursement/tableau-recapitulatif-taux-remboursement |

^a^Source: real cost estimated with the claim database of the study 2014-2019 = total price per domain/quantity.

^b^Source: real cost estimated with the claim database of the study 2014-2019 = total price per domain/quantity.

ATC, anatomical therapeutic chemical; GP, general practitioner.
