# Supplementary figures and images for: Economic and caregiver impact of Alzheimer’s disease across the disease spectrum: a cohort study
Source: Alzheimers Res Ther. 2022 Feb 12;14:34. doi: 10.1186/s13195-022-00969-x (PMC8841058; doi:10.1186/s13195-022-00969-x)

**Supplementary Figure 1:** Flow chart of participant selection


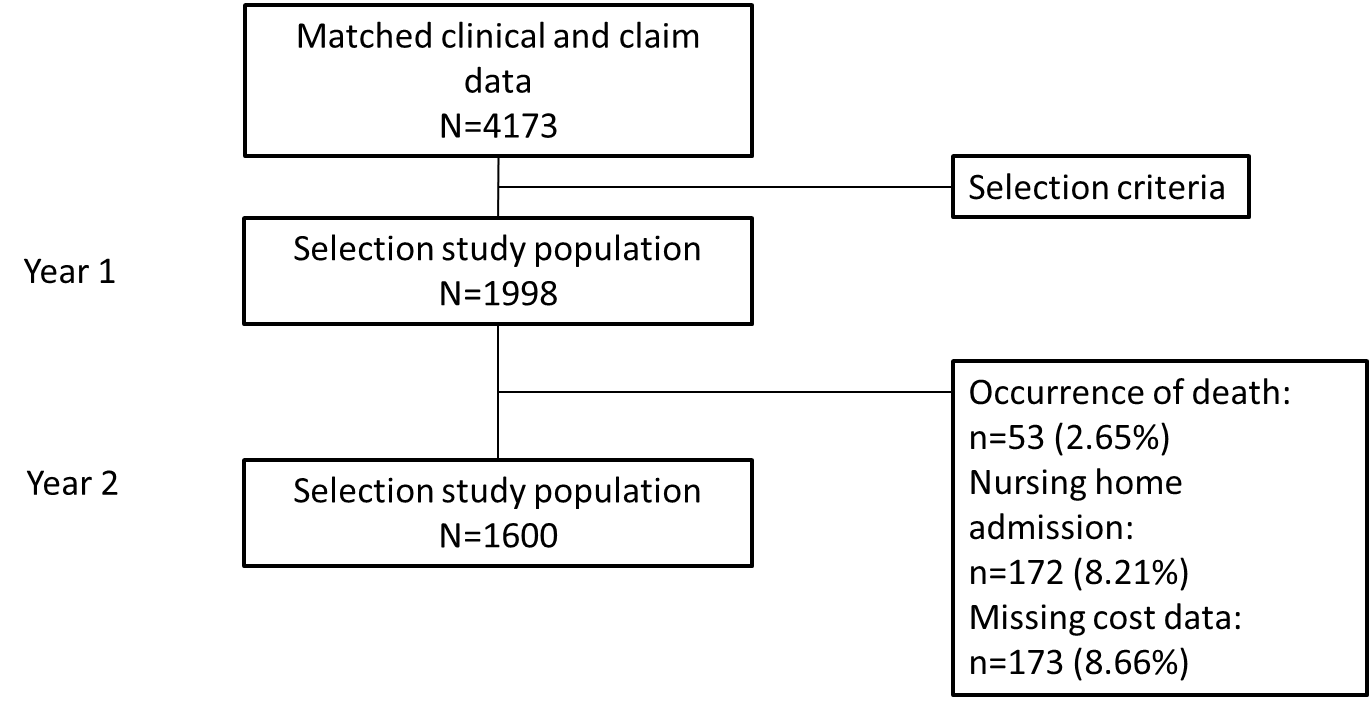

Supplement: Supplementary file 2 — Additional file 2: Figure S1. Flowchart. [file 13195_2022_969_MOESM2_ESM.doc]
