# Supplementary material for: Economic and caregiver impact of Alzheimer’s disease across the disease spectrum: a cohort study
Source: Alzheimers Res Ther. 2022 Feb 12;14:34. doi: 10.1186/s13195-022-00969-x (PMC8841058; doi:10.1186/s13195-022-00969-x)
Supplement: Supplementary file 3 — Additional file 3: Table S2. Cost by Semester. [file 13195_2022_969_MOESM3_ESM.doc]

**Supplementary Table 2: Costs according to the severity of cognitive deficit**

Costs per semester, before or after the initial memory clinic visit, for each diagnosis

|  | | | **Second semester before** | **First semester before** | **First semester after** | **Second semester after** | **Third semester after** | **Fourth semester after** |
| --- | --- | --- | --- | --- | --- | --- | --- | --- |
| All patients | N | | 1782 | 1912 | 1985 | 1784 | 1595 | 1376 |
|  | Missing, n | | 216 | 86 | 13 | 214 | 403 | 622 |
|  | Total costs, € | Mean | 3270 | 4422 | 4909 | 5140 | 5112 | 4388 |
|  | SD | | 7502 | 10217 | 10413 | 12275 | 10658 | 10334 |
|  | SE | | 178 | 234 | 234 | 291 | 267 | 279 |
|  | Median | | 865 | 1068 | 1870 | 1445 | 1589 | 1388 |
|  | IQR | | 1125 | 1444 | 2083 | 2334 | 2354 | 2143 |
|  | Direct medical costs, € | Mean | 3130 | 4243 | 4728 | 4935 | 4938 | 4265 |
|  | SD | | 7100 | 9801 | 10107 | 12040 | 10432 | 10218 |
|  | SE | | 168 | 224 | 227 | 285 | 261 | 275 |
|  | Median | | 844 | 1049 | 1817 | 1366 | 1490 | 1312 |
|  | IQR | | 1115 | 1409 | 2030 | 2221 | 2296 | 1997 |
|  | Direct non-medical costs, € | Mean | 140 | 179 | 181 | 205 | 175 | 123 |
|  | SD | | 2556 | 2748 | 1770 | 1694 | 1898 | 1204 |
|  | SE | | 152 | 140 | 78 | 78 | 91 | 64 |
|  | Median | | 163 | 163 | 199 | 251 | 207 | 195 |
|  | IQR | | 226 | 236 | 264 | 357 | 237 | 197 |
| SCC | n | | 581 | 615 | 634 | 573 | 510 | 437 |
|  | Missing, n | | 59 | 25 | 6 | 67 | 130 | 203 |
|  | Total costs, € | Mean | 3114 | 4209 | 4578 | 3901 | 3815 | 2821 |
|  | SD | | 7425 | 9597 | 10060 | 8487 | 8236 | 6558 |
|  | SE | | 308 | 387 | 400 | 355 | 365 | 314 |
|  | Median | | 801 | 883 | 1731 | 833 | 923 | 859 |
|  | IQR | | 1017 | 1138 | 1734 | 1296 | 1436 | 1141 |
|  | Direct medical costs, € | Mean | 2869 | 3937 | 4332 | 3674 | 3571 | 2742 |
|  | SD | | 6517 | 8898 | 9681 | 7946 | 7687 | 6415 |
|  | SE | | 270 | 359 | 384 | 332 | 340 | 307 |
|  | Median | | 801 | 854 | 1661 | 815 | 920 | 846 |
|  | IQR | | 979 | 1128 | 1667 | 1228 | 1384 | 1119 |
|  | Direct non-medical costs, € | Mean | 245 | 272 | 247 | 226 | 244 | 79 |
|  | SD | | 3553 | 3616 | 2308 | 2329 | 3342 | 912 |
|  | SE | | 379 | 334 | 190 | 215 | 320 | 105 |
|  | Median | | 188 | 149 | 186 | 278 | 193 | 152 |
|  | IQR | | 421 | 259 | 340 | 436 | 235 | 156 |
| MCI | n | | 567 | 604 | 627 | 561 | 509 | 446 |
|  | Missing, n | | 63 | 26 | 3 | 69 | 121 | 184 |
|  | Total costs, € | Mean | 3342 | 4840 | 4876 | 5510 | 4731 | 4285 |
|  | SD | | 7326 | 11,381 | 11,324 | 12,162 | 9444 | 8315 |
|  | SE | | 308 | 463 | 452 | 513 | 419 | 394 |
|  | Median | | 884 | 1112 | 1763 | 1420 | 1442 | 1407 |
|  | IQR | | 1114 | 1214 | 1890 | 2228 | 2151 | 2404 |
|  | Direct medical costs, € | Mean | 3221 | 4647 | 4685 | 5276 | 4563 | 4107 |
|  | SD | | 7072 | 10,953 | 10,895 | 11,932 | 9256 | 8057 |
|  | SE | | 297 | 446 | 435 | 504 | 410 | 382 |
|  | Median | | 877 | 1081 | 1656 | 1358 | 1373 | 1317 |
|  | IQR | | 1070 | 1181 | 1845 | 2075 | 2033 | 1994 |
|  | Direct non-medical costs, € | Mean | 121 | 192 | 190 | 233 | 167 | 179 |
|  | SD | | 2414 | 3091 | 1994 | 1863 | 1298 | 1840 |
|  | SE | | 244 | 270 | 149 | 141 | 107 | 168 |
|  | Median | | 159 | 204 | 203 | 293 | 237 | 231 |
|  | IQR | | 232 | 304 | 293 | 356 | 325 | 258 |
| Mild AD dementia | n | | 180 | 203 | 211 | 193 | 173 | 153 |
|  | Missing, n | | 32 | 9 | 1 | 19 | 39 | 59 |
|  | Total costs, € | Mean | 3558 | 4906 | 5282 | 5823 | 6950 | 7652 |
|  | SD | | 8026 | 13,063 | 10,335 | 12,489 | 14,193 | 19,763 |
|  | SE | | 598 | 917 | 711 | 899 | 1079 | 1598 |
|  | Median | | 923 | 1381 | 2331 | 2425 | 2689 | 2525 |
|  | IQR | | 1259 | 1688 | 2180 | 2796 | 2644 | 1987 |
|  | Direct medical costs, € | Mean | 3496 | 4827 | 5183 | 5587 | 6796 | 7538 |
|  | SD | | 7927 | 12,890 | 10,211 | 12,312 | 14,077 | 19,713 |
|  | SE | | 591 | 905 | 703 | 886 | 1070 | 1594 |
|  | Median | | 906 | 1335 | 2201 | 2395 | 2689 | 2383 |
|  | IQR | | 1159 | 1717 | 2158 | 2537 | 2659 | 1968 |
|  | Direct non-medical costs, € | Mean | 62 | 79 | 99 | 237 | 154 | 114 |
|  | SD | | 517 | 653 | 584 | 848 | 729 | 510 |
|  | SE | | 94 | 106 | 77 | 106 | 103 | 72 |
|  | Median | | 141 | 195 | 173 | 264 | 260 | 175 |
|  | IQR | | 99 | 221 | 244 | 584 | 316 | 174 |
| Moderate AD dementia | n | | 224 | 238 | 254 | 229 | 208 | 174 |
|  | Missing, n | | 32 | 18 | 2 | 27 | 48 | 82 |
|  | Total costs, € | Mean | 3094 | 3271 | 5443 | 5750 | 6041 | 4472 |
|  | SD | | 7808 | 5641 | 10,118 | 13,644 | 10,335 | 7706 |
|  | SE | | 522 | 366 | 635 | 902 | 717 | 584 |
|  | Median | | 810 | 1082 | 2010 | 2232 | 2219 | 2002 |
|  | IQR | | 1377 | 1659 | 2748 | 2817 | 3035 | 2358 |
|  | Direct medical costs, € | Mean | 3057 | 3200 | 5303 | 5583 | 5932 | 4372 |
|  | SD | | 7773 | 5462 | 9988 | 13,528 | 10,225 | 7658 |
|  | SE | | 519 | 354 | 627 | 894 | 709 | 581 |
|  | Median | | 790 | 1045 | 1987 | 2154 | 2168 | 1943 |
|  | IQR | | 1374 | 1661 | 2720 | 2822 | 3173 | 2405 |
|  | Direct non-medical costs, € | Mean | 37 | 71 | 139 | 167 | 109 | 101 |
|  | SD | | 308 | 719 | 806 | 854 | 563 | 508 |
|  | SE | | 52 | 105 | 96 | 106 | 71 | 79 |
|  | Median | | 112 | 84 | 231 | 181 | 203 | 257 |
|  | IQR | | 130 | 140 | 221 | 281 | 151 | 292 |
| Moderately severe/severe AD dementia | n | | 230 | 252 | 259 | 228 | 195 | 166 |
|  | Missing, n | | 30 | 8 | 1 | 32 | 65 | 94 |
|  | Total costs, € | Mean | 3434 | 4637 | 4969 | 6153 | 6882 | 5693 |
|  | SD | | 7448 | 9430 | 9296 | 17,667 | 14,742 | 12,233 |
|  | SE | | 491 | 594 | 578 | 1170 | 1056 | 949 |
|  | Median | | 916 | 1225 | 2274 | 2050 | 2232 | 1875 |
|  | IQR | | 1455 | 2406 | 3129 | 3209 | 3825 | 3464 |
|  | Direct medical costs, € | Mean | 3349 | 4536 | 4865 | 6061 | 6782 | 5571 |
|  | SD | | 7248 | 9250 | 9167 | 17,606 | 14,650 | 12,201 |
|  | SE | | 478 | 583 | 570 | 1166 | 1049 | 947 |
|  | Median | | 899 | 1205 | 2259 | 1950 | 2232 | 1789 |
|  | IQR | | 1455 | 2343 | 3109 | 3214 | 3932 | 3348 |
|  | Direct non-medical costs, € | Mean | 85 | 101 | 104 | 92 | 100 | 122 |
|  | SD | | 1659 | 1046 | 727 | 583 | 403 | 419 |
|  | SE | | 303 | 141 | 91 | 77 | 52 | 53 |
|  | Median | | 201 | 157 | 215 | 200 | 161 | 200 |
|  | IQR | | 246 | 197 | 220 | 163 | 158 | 118 |
| P value* | | | .73 | .005 | .38 | < .0001 | < .0001 | < .0001 |
| P value^†^ | | | .34 | .06 | .47 | .008 | < .0001 | < .0001 |

*GLM comparing mean total costs between diagnosis groups.

^†^GLM adjusted for age, sex, and education level.

AD, Alzheimer’s disease; GLM, general linear model; IQR, interquartile range; MCI, mild cognitive impairment; SCC, subjective cognitive complaint; SD, standard deviation; SE, standard error of the mean.
