# Supplementary material for: Economic and caregiver impact of Alzheimer’s disease across the disease spectrum: a cohort study
Source: Alzheimers Res Ther. 2022 Feb 12;14:34. doi: 10.1186/s13195-022-00969-x (PMC8841058; doi:10.1186/s13195-022-00969-x)
Supplement: Supplementary file 4 — Additional file 4: Table S3. Cost by Domain 1. [file 13195_2022_969_MOESM4_ESM.doc]

**Supplementary Table 3:** **Medical costs per semester for each diagnosis**

|  |  | **Second semester before** | **First semester before** | **First semester after** | **Second semester after** | **Third semester after** | **Fourth semester after** | |
| --- | --- | --- | --- | --- | --- | --- | --- | --- |
| **Ambulatory medicine, €** | | | | | | | | |
| Total direct medical costs | Mean | 726 | 852 | 786 | 613 | 573 | 458 | |
|  | SD | 2772 | 2927 | 2358 | 1961 | 1644 | 1267 | |
|  | SE | 66 | 68 | 53 | 47 | 42 | 35 | |
|  | Median | 292 | 346 | 383 | 267 | 270 | 250 | |
|  | IQR | 277 | 302 | 333 | 291 | 263 | 244 | |
| SCC | Mean | 1119 | 1230 | 1120 | 783 | 806 | 476 | |
|  | SD | 4114 | 4122 | 3287 | 2708 | 2581 | 731 | |
|  | SE | 172 | 167 | 131 | 115 | 115 | 35 | |
|  | Median | 347 | 398 | 453 | 279 | 292 | 293 | |
|  | IQR | 271 | 319 | 358 | 292 | 291 | 269 | |
| MCI | Mean | 711 | 889 | 802 | 638 | 527 | 565 | |
|  | SD | 2397 | 2945 | 2448 | 2049 | 1129 | 2041 | |
|  | SE | 102 | 121 | 98 | 87 | 51 | 98 | |
|  | Median | 312 | 367 | 400 | 310 | 284 | 271 | |
|  | IQR | 305 | 327 | 364 | 334 | 263 | 262 | |
| Mild AD dementia | Mean | 387 | 434 | 471 | 522 | 481 | 377 | |
|  | SD | 576 | 557 | 556 | 702 | 705 | 503 | |
|  | SE | 44 | 40 | 38 | 51 | 54 | 41 | |
|  | Median | 231 | 278 | 319 | 242 | 280 | 227 | |
|  | IQR | 265 | 275 | 227 | 344 | 268 | 225 | |
| Moderate AD dementia | Mean | 334 | 477 | 492 | 436 | 400 | 322 | |
|  | SD | 413 | 740 | 641 | 688 | 589 | 420 | |
|  | SE | 28 | 49 | 41 | 46 | 42 | 32 | |
|  | Median | 218 | 260 | 309 | 219 | 218 | 177 | |
|  | IQR | 223 | 269 | 307 | 248 | 238 | 153 | |
| Moderately severe/severe AD dementia | Mean | 415 | 528 | 479 | 374 | 352 | 339 | |
|  | SD | 949 | 919 | 660 | 523 | 418 | 424 | |
|  | SE | 64 | 59 | 41 | 35 | 30 | 34 | |
|  | Median | 226 | 315 | 301 | 225 | 234 | 207 | |
|  | IQR | 234 | 259 | 241 | 235 | 222 | 272 | |
| P value* |  | < .0001 | < .0001 | < .0001 | < .0001 | < .0001 | < .0001 | |
| P value^†^ |  | < .0001 | < .0001 | < .0001 | < .0001 | < .0001 | < .0001 | |
| **Paramedical medicine, €** | | | | | | | | |
| Total direct medical cost | Mean | 549 | 733 | 1000 | 1094 | 1161 | 1185 | |
|  | SD | 1800 | 1832 | 2107 | 2000 | 1986 | 2002 | |
|  | SE | 54 | 50 | 56 | 55 | 58 | 63 | |
|  | Median | 184 | 250 | 454 | 650 | 740 | 760 | |
|  | IQR | 419 | 578 | 981 | 1295 | 1343 | 1411 | |
| SCC | Mean | 328 | 398 | 537 | 593 | 638 | 709 | |
|  | SD | 1520 | 1523 | 1699 | 1674 | 1614 | 1708 | |
|  | SE | 81 | 75 | 81 | 85 | 86 | 99 | |
|  | Median | 120 | 121 | 199 | 213 | 255 | 274 | |
|  | IQR | 260 | 256 | 314 | 370 | 504 | 618 | |
| MCI | Mean | 487 | 628 | 809 | 909 | 1010 | 1157 | |
|  | SD | 1626 | 1691 | 1982 | 1795 | 1856 | 1990 | |
|  | SE | 85 | 82 | 94 | 89 | 94 | 108 | |
|  | Median | 152 | 235 | 358 | 557 | 618 | 704 | |
|  | IQR | 313 | 460 | 730 | 1020 | 1150 | 1359 | |
| Mild AD dementia | Mean | 695 | 1031 | 1589 | 1676 | 1955 | 1633 | |
|  | SD | 1992 | 1930 | 2462 | 2069 | 2254 | 1869 | |
|  | SE | 196 | 160 | 193 | 170 | 190 | 170 | |
|  | Median | 373 | 712 | 1145 | 1535 | 1768 | 1613 | |
|  | IQR | 811 | 1242 | 1534 | 1724 | 1677 | 1658 | |
| Moderate AD dementia | Mean | 740 | 1093 | 1552 | 1693 | 1656 | 1638 | |
|  | SD | 1865 | 1998 | 2237 | 2085 | 2046 | 1967 | |
|  | SE | 161 | 150 | 165 | 155 | 160 | 171 | |
|  | Median | 279 | 477 | 1531 | 1650 | 1672 | 1599 | |
|  | IQR | 1157 | 1157 | 2055 | 1926 | 1523 | 2242 | |
| Moderately severe/severe AD dementia | Mean | 956 | 1222 | 1576 | 1719 | 1692 | 1622 | |
|  | SD | 2340 | 2190 | 2218 | 2371 | 2159 | 2431 | |
|  | SE | 199 | 163 | 159 | 177 | 177 | 217 | |
|  | Median | 571 | 565 | 1356 | 1346 | 1460 | 1181 | |
|  | IQR | 1309 | 2023 | 2370 | 2196 | 2334 | 2130 | |
| P value* |  | < .0001 | < .0001 | < .0001 | < .0001 | < .0001 | < .0001 | |
| P value^†^ |  | < .0001 | < .0001 | < .0001 | < .0001 | < .0001 | < .0001 | |
| **Pharmaceutical treatment , €** | | | | | | | |  |
| Total direct medical cost | Mean | 462 | 473 | 456 | 449 | 434 | 405 | |
|  | SD | 1376 | 1638 | 1436 | 1060 | 1269 | 1291 | |
|  | SE | 34 | 39 | 33 | 26 | 33 | 36 | |
|  | Median | 212 | 220 | 254 | 252 | 249 | 254 | |
|  | IQR | 256 | 264 | 300 | 265 | 242 | 252 | |
| SCC | Mean | 492 | 492 | 415 | 399 | 400 | 345 | |
|  | SD | 1214 | 1300 | 854 | 750 | 979 | 785 | |
|  | SE | 52 | 54 | 35 | 32 | 45 | 39 | |
|  | Median | 184 | 174 | 186 | 185 | 190 | 181 | |
|  | IQR | 247 | 253 | 255 | 219 | 204 | 252 | |
| MCI | Mean | 441 | 432 | 445 | 489 | 437 | 404 | |
|  | SD | 915 | 807 | 905 | 1098 | 789 | 621 | |
|  | SE | 40 | 34 | 38 | 48 | 36 | 31 | |
|  | Median | 224 | 229 | 245 | 263 | 272 | 270 | |
|  | IQR | 249 | 253 | 298 | 284 | 262 | 262 | |
| Mild AD dementia | Mean | 526 | 603 | 626 | 478 | 449 | 458 | |
|  | SD | 3266 | 4159 | 3702 | 1877 | 1329 | 1567 | |
|  | SE | 261 | 303 | 264 | 139 | 103 | 133 | |
|  | Median | 233 | 258 | 303 | 271 | 295 | 309 | |
|  | IQR | 268 | 242 | 266 | 232 | 174 | 228 | |
| Moderate AD dementia | Mean | 376 | 425 | 424 | 461 | 557 | 596 | |
|  | SD | 617 | 786 | 595 | 1011 | 2624 | 2882 | |
|  | SE | 43 | 53 | 39 | 70 | 188 | 228 | |
|  | Median | 197 | 242 | 335 | 322 | 273 | 300 | |
|  | IQR | 250 | 264 | 312 | 309 | 261 | 246 | |
| Moderately severe/severe AD dementia | Mean | 471 | 468 | 475 | 440 | 368 | 319 | |
|  | SD | 819 | 700 | 688 | 651 | 420 | 435 | |
|  | SE | 55 | 45 | 44 | 44 | 31 | 36 | |
|  | Median | 246 | 267 | 317 | 323 | 280 | 252 | |
|  | IQR | 266 | 305 | 293 | 260 | 194 | 169 | |
| P value* |  | .016 | .004 | .001 | .026 | .005 | < .0001 | |
| P value^†^ |  | .035 | .012 | .001 | .031 | .003 | < .0001 | |
| **Stays in public hospitals, €** | | | | | | | | |
| Total direct medical cost | Mean | 1292 | 2032 | 2424 | 2737 | 2699 | 2188 | |
|  | SD | 13,282 | 18,125 | 14,640 | 23,172 | 18,800 | 20,903 | |
|  | SE | 866 | 1072 | 566 | 1304 | 1142 | 1453 | |
|  | Median | 4077 | 6777 | 1501 | 7309 | 9228 | 6468 | |
|  | IQR | 6975 | 9750 | 1636 | 9444 | 14,283 | 10,615 | |
| SCC | Mean | 730 | 1628 | 2133 | 1672 | 1553 | 1010 | |
|  | SD | 12,029 | 14,591 | 13,824 | 12,415 | 13,694 | 12,778 | |
|  | SE | 1718 | 1547 | 912 | 1301 | 1614 | 1844 | |
|  | Median | 3948 | 5147 | 1359 | 5944 | 5850 | 4991 | |
|  | IQR | 3509 | 9762 | 1374 | 9480 | 8106 | 5442 | |
| MCI | Mean | 1524 | 2565 | 2574 | 3298 | 2568 | 2057 | |
|  | SD | 12,783 | 20,736 | 15,015 | 20,671 | 15,877 | 13,868 | |
|  | SE | 1412 | 2198 | 973 | 1903 | 1646 | 1580 | |
|  | Median | 4325 | 10,297 | 2169 | 9183 | 8240 | 5095 | |
|  | IQR | 8582 | 10,994 | 1479 | 7524 | 11,565 | 10,434 | |
| Mild AD dementia | Mean | 1787 | 2744 | 2572 | 3078 | 3977 | 5117 | |
|  | SD | 11,649 | 23,328 | 14,301 | 21,843 | 25,768 | 37,965 | |
|  | SE | 1969 | 3688 | 1816 | 3256 | 4419 | 6931 | |
|  | Median | 3066 | 6562 | 2142 | 3045 | 9727 | 12,723 | |
|  | IQR | 7364 | 9388 | 3755 | 9675 | 19,767 | 24,282 | |
| Moderate AD dementia | Mean | 1551 | 1077 | 2791 | 2999 | 3209 | 1842 | |
|  | SD | 16,586 | 8899 | 15,515 | 28,468 | 14,980 | 13,530 | |
|  | SE | 2804 | 1682 | 1841 | 4812 | 2463 | 2821 | |
|  | Median | 4453 | 4896 | 1421 | 7357 | 16,620 | 11,217 | |
|  | IQR | 4960 | 13,252 | 3720 | 9804 | 18,138 | 10,046 | |
| Moderately severe/severe AD dementia | Mean | 1496 | 2072 | 2291 | 3477 | 4366 | 3308 | |
|  | SD | 14,572 | 17,055 | 15,137 | 43,169 | 26,343 | 23,459 | |
|  | SE | 2499 | 2697 | 1836 | 8308 | 4453 | 4356 | |
|  | Median | 3795 | 7683 | 2313 | 23,674 | 18,393 | 10,706 | |
|  | IQR | 4310 | 6893 | 3572 | 20,434 | 11,350 | 9474 | |
| P value* |  | .901 | .127 | .004 | < .0001 | .002 | .001 | |
| P value^†^ |  | .838 | .205 | .004 | .002 | .008 | .004 | |
| **Stays in private hospitals , €** | | | | | | | | |
| Total direct medical cost | Mean | 243 | 331 | 243 | 247 | 245 | 152 | |
|  | SD | 5096 | 5664 | 5488 | 5441 | 4915 | 3843 | |
|  | SE | 419 | 416 | 457 | 458 | 429 | 410 | |
|  | Median | 1138 | 1015 | 1012 | 1123 | 1438 | 1093 | |
|  | IQR | 922 | 1803 | 1431 | 1109 | 1168 | 956 | |
| SCC | Mean | 445 | 460 | 374 | 454 | 418 | 280 | |
|  | SD | 7168 | 6551 | 6050 | 7183 | 6853 | 5954 | |
|  | SE | 925 | 762 | 745 | 935 | 989 | 1069 | |
|  | Median | 1498 | 965 | 1010 | 1123 | 1494 | 1474 | |
|  | IQR | 1474 | 937 | 1043 | 1596 | 1377 | 1567 | |
| MCI | Mean | 179 | 325 | 245 | 176 | 188 | 104 | |
|  | SD | 2395 | 4769 | 5448 | 3067 | 2712 | 1278 | |
|  | SE | 342 | 616 | 812 | 452 | 400 | 216 | |
|  | Median | 1451 | 1243 | 1015 | 1109 | 1146 | 768 | |
|  | IQR | 1027 | 2235 | 1926 | 1038 | 863 | 682 | |
| Mild AD dementia | Mean | 162 | 94 | 25 | 69 | 88 | 67 | |
|  | SD | 4361 | 1781 | 495 | 1043 | 1308 | 771 | |
|  | SE | 1315 | 494 | 175 | 348 | 394 | 273 | |
|  | Median | 1110 | 767 | 455 | 1438 | 725 | 1258 | |
|  | IQR | 525 | 810 | 347 | 850 | 1755 | 622 | |
| Moderate AD dementia | Mean | 93 | 199 | 184 | 160 | 220 | 74 | |
|  | SD | 2755 | 6199 | 6218 | 6892 | 5362 | 1571 | |
|  | SE | 764 | 1657 | 1875 | 2179 | 1433 | 594 | |
|  | Median | 659 | 1738 | 2197 | 1276 | 1508 | 1451 | |
|  | IQR | 290 | 2675 | 3469 | 2327 | 1603 | 940 | |
| Moderately severe/severe AD dementia | Mean | 96 | 347 | 148 | 143 | 103 | 106 | |
|  | SD | 1284 | 5993 | 3467 | 2496 | 1415 | 1820 | |
|  | SE | 331 | 1223 | 927 | 605 | 409 | 688 | |
|  | Median | 824 | 1585 | 1444 | 1430 | 1327 | 1962 | |
|  | IQR | 862 | 2230 | 1976 | 1307 | 638 | 2622 | |
| P value* |  | < .0001 | .125 | .002 | .002 | .096 | < .0001 | |
| P value^†^ |  | .283 | .07 | .001 | .321 | .117 | .001 | |

*GLM comparing mean total medical costs between diagnosis groups.

^†^GLM adjusted for age, sex, and education level.

AD, Alzheimer’s disease; GLM, general linear model; IQR, interquartile range; MCI, mild cognitive impairment; SCC, subjective cognitive complaint; SD, standard deviation; SE, standard error of the mean.
