# Supplementary material for: Economic and caregiver impact of Alzheimer’s disease across the disease spectrum: a cohort study
Source: Alzheimers Res Ther. 2022 Feb 12;14:34. doi: 10.1186/s13195-022-00969-x (PMC8841058; doi:10.1186/s13195-022-00969-x)
Supplement: Supplementary file 5 — Additional file 5: Table S4. Cost by Domain 2. [file 13195_2022_969_MOESM5_ESM.doc]

**Supplementary Table 4: Costs per semester for ambulatory medicine, paramedical medicine, pharmaceutical treatment, and public hospital stays by diagnosis**

|  |  | **Second semester before** | **First semester before** | **First semester after** | **Second semester after** | **Third semester after** | **Fourth semester after** |
| --- | --- | --- | --- | --- | --- | --- | --- |
| **Ambulatory medicine, €** | | | | | | | |
| **General practitioner** | | | | | | | |
| Total | Mean | 70 | 94 | 80 | 69 | 71 | 73 |
|  | SD | 73 | 95 | 94 | 72 | 75 | 80 |
|  | SE | 2 | 2 | 2 | 2 | 2 | 2 |
|  | Median | 64 | 69 | 67 | 61 | 65 | 66 |
|  | IQR | 45 | 52 | 48 | 37 | 40 | 39 |
| SCC | Mean | 72 | 82 | 79 | 65 | 65 | 71 |
|  | SD | 73 | 84 | 92 | 70 | 73 | 75 |
|  | SE | 3 | 4 | 4 | 3 | 3 | 4 |
|  | Median | 63 | 69 | 63 | 50 | 50 | 59 |
|  | IQR | 46 | 49 | 49 | 36 | 37 | 42 |
| MCI | Mean | 73 | 89 | 77 | 69 | 74 | 80 |
|  | SD | 71 | 91 | 78 | 65 | 77 | 91 |
|  | SE | 3 | 4 | 3 | 3 | 4 | 5 |
|  | Median | 67 | 69 | 67 | 67 | 69 | 67 |
|  | IQR | 46 | 50 | 49 | 39 | 44 | 37 |
| Mild AD | Mean | 63 | 84 | 72 | 69 | 64 | 63 |
|  | SD | 64 | 103 | 82 | 73 | 54 | 69 |
|  | SE | 5 | 8 | 6 | 6 | 4 | 6 |
|  | Median | 57 | 67 | 69 | 67 | 56 | 65 |
|  | IQR | 34 | 49 | 37 | 43 | 32 | 34 |
| Moderate AD | Mean | 63 | 94 | 81 | 69 | 72 | 71 |
|  | SD | 69 | 108 | 113 | 65 | 71 | 70 |
|  | SE | 5 | 7 | 8 | 5 | 5 | 6 |
|  | Median | 62 | 72 | 63 | 61 | 67 | 69 |
|  | IQR | 41 | 59 | 36 | 42 | 45 | 46 |
| Moderately severe/severe AD | Mean | 73 | 96 | 92 | 82 | 79 | 74 |
|  | SD | 88 | 106 | 119 | 98 | 91 | 78 |
|  | SE | 6 | 7 | 8 | 7 | 7 | 7 |
|  | Median | 68 | 79 | 73 | 68 | 69 | 60 |
|  | IQR | 46 | 61 | 52 | 46 | 44 | 56 |
| **Geriatrician** | | | | | | | |
| Total | Mean | 1 | 2 | 16 | 3 | 5 | 3 |
|  | SD | 35 | 45 | 38 | 37 | 171 | 30 |
|  | SE | 7 | 5 | 2 | 3 | 13 | 3 |
|  | Median | 40 | 28 | 46 | 26 | 26 | 23 |
|  | IQR | 24 | 24 | 24 | 4 | 4 | 4 |
| SCC | Mean | 1 | 2 | 14 | 2 | 4 | 2 |
|  | SD | 28 | 16 | 30 | 73 | 136 | 12 |
|  | SE | 10 | 3 | 2 | 13 | 21 | 2 |
|  | Median | 34 | 23 | 38 | 23 | 23 | 23 |
|  | IQR | 31 | 6 | 22 | 8 | 12 | 6 |
| MCI | Mean | 1 | 3 | 17 | 3 | 5 | 3 |
|  | SD | 18 | 33 | 42 | 14 | 41 | 16 |
|  | SE | 6 | 5 | 3 | 2 | 5 | 2 |
|  | Median | 40 | 34 | 47 | 26 | 27 | 23 |
|  | IQR | 22 | 36 | 22 | 5 | 5 | 4 |
| Mild AD | Mean | 1 | 1 | 13 | 2 | 16 | 4 |
|  | SD | 62 | 54 | 31 | 8 | 404 | 72 |
|  | SE | 44 | 24 | 4 | 2 | 79 | 21 |
|  | Median | 76 | 45 | 49 | 23 | 23 | 26 |
|  | IQR |  | 31 | 34 | 3 | 4 | 5 |
| Moderate AD | Mean | 1 | 4 | 16 | 4 | 5 | 2 |
|  | SD | 71 | 94 | 35 | 16 | 71 | 8 |
|  | SE | 41 | 27 | 4 | 3 | 15 | 2 |
|  | Median | 46 | 48 | 49 | 27 | 26 | 23 |
|  | IQR | 89 | 53 | 43 | 13 | 4 | 4 |
| Moderately severe/severe AD | Mean | 1 | 1 | 19 | 4 | 2 | 5 |
|  | SD | 30 | 35 | 45 | 27 | 12 | 28 |
|  | SE | 17 | 16 | 5 | 5 | 3 | 7 |
|  | Median | 35 | 47 | 45 | 26 | 27 | 38 |
|  | IQR | 39 | 17 | 26 | 6 | 4 | 24 |
| **Neurologist** | | | | | | | |
| Total | Mean | 3 | 4 | 7 | 3 | 4 | 3 |
|  | SD | 27 | 47 | 33 | 33 | 30 | 30 |
|  | SE | 3 | 4 | 2 | 4 | 3 | 3 |
|  | Median | 50 | 43 | 62 | 59 | 60 | 59 |
|  | IQR | 18 | 18 | 23 | 19 | 21 | 20 |
| SCC | Mean | 3 | 5 | 8 | 3 | 4 | 5 |
|  | SD | 32 | 49 | 31 | 51 | 38 | 41 |
|  | SE | 5 | 8 | 3 | 10 | 6 | 7 |
|  | Median | 43 | 59 | 49 | 59 | 60 | 50 |
|  | IQR | 18 | 19 | 19 | 17 | 7 | 18 |
| MCI | Mean | 3 | 5 | 8 | 3 | 3 | 3 |
|  | SD | 22 | 47 | 30 | 25 | 27 | 15 |
|  | SE | 4 | 6 | 4 | 4 | 5 | 3 |
|  | Median | 42 | 41 | 63 | 59 | 59 | 59 |
|  | IQR | 19 | 18 | 22 | 20 | 11 | 20 |
| Mild AD | Mean | 4 | 2 | 6 | 3 | 3 | 5 |
|  | SD | 17 | 13 | 55 | 18 | 25 | 27 |
|  | SE | 5 | 4 | 15 | 6 | 8 | 8 |
|  | Median | 52 | 43 | 60 | 52 | 55 | 59 |
|  | IQR | 19 | 19 | 23 | 20 | 30 | 18 |
| Moderate AD | Mean | 4 | 5 | 6 | 3 | 5 | 3 |
|  | SD | 34 | 65 | 36 | 16 | 23 | 16 |
|  | SE | 10 | 18 | 8 | 5 | 6 | 6 |
|  | Median | 59 | 59 | 70 | 62 | 68 | 59 |
|  | IQR | 31 | 66 | 25 | 8 | 13 | 28 |
| Moderately severe/severe AD | Mean | 3 | 2 | 6 | 2 | 2 | 3 |
|  | SD | 16 | 16 | 29 | 18 | 12 | 23 |
|  | SE | 5 | 5 | 6 | 6 | 4 | 9 |
|  | Median | 60 | 42 | 69 | 40 | 58 | 69 |
|  | IQR | 13 | 17 | 25 | 24 | 16 | 5 |
| **Psychiatrist** | | | | | | | |
| Total | Mean | 6 | 9 | 8 | 5 | 8 | 4 |
|  | SD | 178 | 309 | 355 | 219 | 502 | 150 |
|  | SE | 22 | 34 | 40 | 28 | 64 | 24 |
|  | Median | 85 | 83 | 61 | 79 | 54 | 102 |
|  | IQR | 143 | 120 | 71 | 85 | 80 | 107 |
| SCC | Mean | 11 | 14 | 14 | 11 | 11 | 6 |
|  | SD | 194 | 303 | 358 | 253 | 324 | 131 |
|  | SE | 34 | 46 | 55 | 43 | 56 | 29 |
|  | Median | 141 | 105 | 84 | 116 | 64 | 111 |
|  | IQR | 130 | 116 | 107 | 73 | 75 | 53 |
| MCI | Mean | 5 | 8 | 9 | 4 | 5 | 5 |
|  | SD | 148 | 344 | 436 | 195 | 201 | 167 |
|  | SE | 34 | 70 | 91 | 46 | 49 | 46 |
|  | Median | 82 | 54 | 65 | 48 | 61 | 74 |
|  | IQR | 69 | 145 | 49 | 45 | 179 | 181 |
| Mild AD | Mean | 5 | 6 | 3 | 3 | 21 | 5 |
|  | SD | 120 | 194 | 143 | 111 | 1641 | 168 |
|  | SE | 53 | 79 | 64 | 56 | 820 | 119 |
|  | Median | 197 | 144 | 61 | 93 | 142 | 360 |
|  | IQR | 154 | 150 | 197 | 145 | 1249 |  |
| Moderate AD | Mean | 4 | 8 | 1 | 1 | 5 | 0 |
|  | SD | 247 | 415 | 15 | 21 | 563 |  |
|  | SE | 101 | 169 | 7 | 10 | 325 |  |
|  | Median | 42 | 82 | 29 | 42 | 29 |  |
|  | IQR | 71 | 536 | 1 | 14 | 660 |  |
| Moderately severe/severe AD | Mean | 0 | 1 | 1 | 1 | 1 | 0 |
|  | SD | 1 | 5 | 9 | 103 | 24 | 5 |
|  | SE | 1 | 3 | 4 | 73 | 14 | 3 |
|  | Median | 31 | 40 | 42 | 115 | 42 | 41 |
|  | IQR |  | 6 | 6 |  | 28 |  |
| **Other ambulatory costs** | | | | | | | |
| Total | Mean | 645 | 749 | 676 | 532 | 485 | 374 |
|  | SD | 2836 | 2980 | 2389 | 2017 | 1677 | 1291 |
|  | SE | 70 | 70 | 55 | 50 | 44 | 36 |
|  | Median | 225 | 274 | 295 | 213 | 208 | 188 |
|  | IQR | 240 | 270 | 283 | 261 | 232 | 217 |
| SCC | Mean | 1032 | 1127 | 1004 | 702 | 721 | 393 |
|  | SD | 4192 | 4189 | 3339 | 2783 | 2625 | 728 |
|  | SE | 178 | 173 | 135 | 121 | 119 | 36 |
|  | Median | 270 | 315 | 355 | 230 | 224 | 211 |
|  | IQR | 239 | 292 | 321 | 263 | 247 | 231 |
| MCI | Mean | 630 | 785 | 692 | 559 | 439 | 475 |
|  | SD | 2437 | 2988 | 2469 | 2083 | 1144 | 2089 |
|  | SE | 105 | 125 | 100 | 90 | 53 | 103 |
|  | Median | 244 | 296 | 312 | 234 | 215 | 195 |
|  | IQR | 278 | 300 | 319 | 282 | 258 | 218 |
| Mild AD | Mean | 314 | 340 | 376 | 446 | 377 | 299 |
|  | SD | 570 | 523 | 515 | 707 | 557 | 478 |
|  | SE | 45 | 39 | 36 | 54 | 43 | 40 |
|  | Median | 191 | 196 | 255 | 214 | 208 | 181 |
|  | IQR | 239 | 248 | 209 | 324 | 215 | 212 |
| Moderate AD | Mean | 262 | 366 | 388 | 360 | 313 | 246 |
|  | SD | 383 | 699 | 616 | 690 | 585 | 406 |
|  | SE | 27 | 47 | 40 | 48 | 43 | 33 |
|  | Median | 163 | 194 | 248 | 151 | 155 | 127 |
|  | IQR | 224 | 210 | 280 | 209 | 225 | 160 |
| Moderately severe/severe AD | Mean | 338 | 428 | 361 | 284 | 268 | 258 |
|  | SD | 923 | 874 | 600 | 515 | 409 | 403 |
|  | SE | 65 | 58 | 38 | 37 | 31 | 33 |
|  | Median | 168 | 230 | 224 | 165 | 178 | 134 |
|  | IQR | 195 | 222 | 194 | 192 | 196 | 204 |
| **Paramedical medicine, €** | | | | | | | |
| **Physiotherapists** | | | | | | | |
| Total | Mean | 124 | 146 | 164 | 165 | 185 | 194 |
|  | SD | 494 | 516 | 482 | 459 | 470 | 480 |
|  | SE | 22 | 21 | 19 | 19 | 20 | 22 |
|  | Median | 264 | 295 | 319 | 331 | 373 | 432 |
|  | IQR | 280 | 286 | 358 | 385 | 373 | 485 |
| SCC | Mean | 126 | 148 | 173 | 168 | 182 | 176 |
|  | SD | 475 | 477 | 467 | 473 | 447 | 449 |
|  | SE | 35 | 32 | 30 | 32 | 31 | 35 |
|  | Median | 242 | 252 | 262 | 246 | 291 | 294 |
|  | IQR | 236 | 247 | 245 | 250 | 298 | 484 |
| MCI | Mean | 137 | 156 | 159 | 170 | 178 | 179 |
|  | SD | 550 | 559 | 496 | 484 | 517 | 474 |
|  | SE | 42 | 39 | 34 | 36 | 39 | 38 |
|  | Median | 235 | 294 | 320 | 383 | 365 | 353 |
|  | IQR | 241 | 271 | 338 | 376 | 320 | 388 |
| Mild AD | Mean | 129 | 143 | 202 | 205 | 246 | 261 |
|  | SD | 391 | 401 | 517 | 418 | 449 | 504 |
|  | SE | 58 | 51 | 62 | 51 | 55 | 68 |
|  | Median | 363 | 388 | 519 | 548 | 569 | 661 |
|  | IQR | 385 | 266 | 563 | 422 | 568 | 489 |
| Moderate AD | Mean | 86 | 106 | 142 | 132 | 158 | 190 |
|  | SD | 414 | 413 | 447 | 391 | 371 | 460 |
|  | SE | 66 | 56 | 56 | 49 | 45 | 63 |
|  | Median | 365 | 369 | 377 | 337 | 372 | 482 |
|  | IQR | 526 | 446 | 432 | 318 | 370 | 511 |
| Moderately severe/severe AD | Mean | 118 | 155 | 143 | 146 | 186 | 224 |
|  | SD | 501 | 650 | 470 | 430 | 490 | 506 |
|  | SE | 68 | 81 | 55 | 53 | 63 | 71 |
|  | Median | 345 | 373 | 397 | 355 | 504 | 742 |
|  | IQR | 350 | 268 | 386 | 499 | 349 | 510 |
| **Nurses** | | | | | | | |
| Total | Mean | 389 | 540 | 760 | 840 | 880 | 893 |
|  | SD | 1776 | 1773 | 2079 | 1986 | 1960 | 1980 |
|  | SE | 61 | 53 | 62 | 61 | 63 | 69 |
|  | Median | 25 | 33 | 189 | 309 | 363 | 366 |
|  | IQR | 151 | 337 | 1227 | 1591 | 1745 | 1781 |
| SCC | Mean | 184 | 226 | 325 | 390 | 403 | 473 |
|  | SD | 1537 | 1493 | 1711 | 1686 | 1591 | 1707 |
|  | SE | 95 | 85 | 95 | 100 | 100 | 114 |
|  | Median | 12 | 14 | 16 | 18 | 20 | 26 |
|  | IQR | 25 | 33 | 76 | 110 | 152 | 245 |
| MCI | Mean | 318 | 430 | 581 | 644 | 729 | 870 |
|  | SD | 1543 | 1514 | 1874 | 1681 | 1765 | 1932 |
|  | SE | 92 | 80 | 102 | 92 | 100 | 114 |
|  | Median | 27 | 24 | 133 | 154 | 264 | 250 |
|  | IQR | 69 | 175 | 817 | 1112 | 1205 | 1397 |
| Mild AD | Mean | 505 | 775 | 1233 | 1283 | 1505 | 1168 |
|  | SD | 2074 | 1969 | 2416 | 2106 | 2216 | 1844 |
|  | SE | 233 | 175 | 205 | 191 | 204 | 181 |
|  | Median | 65 | 169 | 928 | 1723 | 1694 | 1243 |
|  | IQR | 402 | 1139 | 2031 | 2088 | 2487 | 1868 |
| Moderate AD | Mean | 582 | 918 | 1292 | 1432 | 1373 | 1339 |
|  | SD | 1802 | 1937 | 2196 | 1999 | 2024 | 1939 |
|  | SE | 169 | 155 | 174 | 159 | 168 | 186 |
|  | Median | 186 | 378 | 1542 | 1656 | 1755 | 1948 |
|  | IQR | 1134 | 1358 | 1990 | 1886 | 1959 | 2404 |
| Moderately severe/severe AD | Mean | 804 | 1021 | 1349 | 1484 | 1440 | 1342 |
|  | SD | 2200 | 2108 | 2165 | 2395 | 2147 | 2401 |
|  | SE | 200 | 167 | 167 | 192 | 194 | 237 |
|  | Median | 267 | 418 | 1618 | 1435 | 1796 | 1405 |
|  | IQR | 1885 | 2184 | 2642 | 3033 | 2761 | 2868 |
| **Other paramedics** | | | | | | | |
| Total | Mean | 36 | 48 | 75 | 86 | 95 | 95 |
|  | SD | 618 | 582 | 621 | 532 | 531 | 517 |
|  | SE | 53 | 42 | 37 | 35 | 34 | 37 |
|  | Median | 179 | 252 | 329 | 612 | 565 | 627 |
|  | IQR | 426 | 460 | 557 | 420 | 411 | 385 |
| SCC | Mean | 18 | 23 | 38 | 35 | 53 | 61 |
|  | SD | 503 | 646 | 609 | 498 | 577 | 536 |
|  | SE | 75 | 90 | 73 | 69 | 79 | 78 |
|  | Median | 45 | 47 | 70 | 163 | 290 | 445 |
|  | IQR | 48 | 55 | 180 | 428 | 546 | 488 |
| MCI | Mean | 33 | 42 | 67 | 86 | 103 | 108 |
|  | SD | 600 | 571 | 612 | 544 | 513 | 502 |
|  | SE | 96 | 78 | 63 | 62 | 56 | 60 |
|  | Median | 283 | 231 | 235 | 571 | 554 | 699 |
|  | IQR | 460 | 313 | 338 | 365 | 404 | 304 |
| Mild AD | Mean | 61 | 113 | 154 | 187 | 203 | 204 |
|  | SD | 481 | 471 | 473 | 492 | 513 | 544 |
|  | SE | 108 | 85 | 70 | 75 | 77 | 87 |
|  | Median | 481 | 666 | 677 | 759 | 687 | 721 |
|  | IQR | 555 | 370 | 451 | 408 | 364 | 281 |
| Moderate AD | Mean | 76 | 76 | 114 | 124 | 118 | 94 |
|  | SD | 779 | 540 | 706 | 523 | 563 | 431 |
|  | SE | 170 | 102 | 110 | 90 | 95 | 86 |
|  | Median | 593 | 474 | 610 | 767 | 663 | 691 |
|  | IQR | 575 | 621 | 523 | 479 | 495 | 363 |
| Moderately severe/severe AD | Mean | 34 | 46 | 85 | 88 | 66 | 56 |
|  | SD | 687 | 507 | 566 | 435 | 373 | 570 |
|  | SE | 191 | 101 | 109 | 82 | 83 | 147 |
|  | Median | 91 | 275 | 751 | 738 | 639 | 435 |
|  | IQR | 881 | 433 | 339 | 242 | 363 | 462 |
| **Pharmaceutical treatment, €** | | | | | | | |
| **AD medication** | | | | | | | |
| Total | Mean | 25 | 33 | 49 | 52 | 48 | 48 |
|  | SD | 182 | 180 | 174 | 157 | 138 | 139 |
|  | SE | 14 | 11 | 9 | 8 | 7 | 8 |
|  | Median | 230 | 213 | 213 | 213 | 194 | 182 |
|  | IQR | 159 | 152 | 132 | 117 | 107 | 100 |
| SCC | Mean | 2 | 3 | 4 | 9 | 10 | 10 |
|  | SD | 128 | 196 | 100 | 166 | 145 | 147 |
|  | SE | 45 | 69 | 24 | 33 | 27 | 30 |
|  | Median | 59 | 221 | 157 | 167 | 128 | 142 |
|  | IQR | 127 | 291 | 130 | 208 | 129 | 120 |
| MCI | Mean | 14 | 16 | 27 | 40 | 42 | 40 |
|  | SD | 160 | 179 | 171 | 156 | 138 | 147 |
|  | SE | 28 | 28 | 19 | 17 | 15 | 16 |
|  | Median | 227 | 195 | 187 | 239 | 239 | 195 |
|  | IQR | 100 | 89 | 144 | 111 | 96 | 112 |
| Mild AD | Mean | 43 | 59 | 107 | 100 | 98 | 104 |
|  | SD | 128 | 128 | 176 | 174 | 149 | 138 |
|  | SE | 21 | 16 | 18 | 19 | 17 | 16 |
|  | Median | 222 | 184 | 203 | 195 | 174 | 189 |
|  | IQR | 92 | 106 | 77 | 90 | 70 | 96 |
| Moderate AD | Mean | 60 | 83 | 117 | 107 | 87 | 98 |
|  | SD | 163 | 172 | 168 | 141 | 136 | 151 |
|  | SE | 24 | 20 | 16 | 14 | 15 | 18 |
|  | Median | 288 | 236 | 243 | 215 | 196 | 209 |
|  | IQR | 190 | 155 | 189 | 126 | 150 | 141 |
| Moderately severe/severe AD | Mean | 67 | 76 | 98 | 91 | 75 | 67 |
|  | SD | 230 | 215 | 187 | 157 | 119 | 104 |
|  | SE | 33 | 27 | 19 | 17 | 14 | 13 |
|  | Median | 263 | 224 | 230 | 213 | 181 | 150 |
|  | IQR | 196 | 206 | 174 | 117 | 91 | 85 |
| **Psychotics/hypnotics** | | | | | | | |
| Total | Mean | 20 | 21 | 26 | 27 | 26 | 26 |
|  | SD | 91 | 77 | 76 | 77 | 61 | 60 |
|  | SE | 3 | 2 | 2 | 2 | 2 | 2 |
|  | Median | 22 | 25 | 30 | 31 | 32 | 31 |
|  | IQR | 28 | 26 | 31 | 30 | 31 | 28 |
| SCC | Mean | 19 | 20 | 22 | 23 | 19 | 18 |
|  | SD | 137 | 115 | 107 | 105 | 61 | 44 |
|  | SE | 9 | 7 | 6 | 6 | 4 | 3 |
|  | Median | 21 | 23 | 25 | 23 | 23 | 24 |
|  | IQR | 25 | 21 | 28 | 25 | 25 | 22 |
| MCI | Mean | 18 | 18 | 22 | 25 | 25 | 26 |
|  | SD | 55 | 50 | 60 | 69 | 75 | 76 |
|  | SE | 3 | 3 | 3 | 4 | 5 | 5 |
|  | Median | 19 | 24 | 27 | 28 | 30 | 29 |
|  | IQR | 27 | 27 | 28 | 29 | 31 | 29 |
| Mild AD | Mean | 18 | 25 | 33 | 32 | 30 | 30 |
|  | SD | 57 | 55 | 62 | 54 | 42 | 66 |
|  | SE | 7 | 5 | 6 | 5 | 4 | 7 |
|  | Median | 23 | 30 | 39 | 40 | 38 | 38 |
|  | IQR | 30 | 31 | 30 | 29 | 31 | 23 |
| Moderate AD | Mean | 22 | 27 | 32 | 33 | 32 | 33 |
|  | SD | 77 | 69 | 66 | 68 | 55 | 54 |
|  | SE | 8 | 6 | 6 | 6 | 5 | 5 |
|  | Median | 21 | 31 | 45 | 45 | 37 | 40 |
|  | IQR | 36 | 39 | 54 | 32 | 36 | 24 |
| Moderately severe/severe AD | Mean | 22 | 22 | 33 | 33 | 35 | 30 |
|  | SD | 49 | 38 | 50 | 46 | 48 | 39 |
|  | SE | 5 | 3 | 4 | 4 | 4 | 4 |
|  | Median | 27 | 27 | 37 | 38 | 39 | 32 |
|  | IQR | 29 | 30 | 36 | 27 | 30 | 30 |
| **Other pharmaceutical treatments** | | | | | | | |
| Total | Mean | 417 | 420 | 388 | 372 | 362 | 338 |
|  | SD | 1377 | 1641 | 1445 | 1059 | 1258 | 1277 |
|  | SE | 34 | 39 | 34 | 26 | 33 | 36 |
|  | Median | 171 | 171 | 168 | 165 | 162 | 156 |
|  | IQR | 203 | 211 | 216 | 184 | 187 | 184 |
| SCC | Mean | 471 | 470 | 404 | 370 | 365 | 338 |
|  | SD | 1213 | 1301 | 875 | 745 | 916 | 778 |
|  | SE | 52 | 54 | 36 | 32 | 42 | 38 |
|  | Median | 164 | 161 | 163 | 163 | 156 | 156 |
|  | IQR | 229 | 228 | 231 | 194 | 201 | 225 |
| MCI | Mean | 409 | 398 | 406 | 422 | 372 | 333 |
|  | SD | 906 | 801 | 913 | 1101 | 784 | 594 |
|  | SE | 39 | 33 | 38 | 48 | 36 | 29 |
|  | Median | 196 | 195 | 189 | 184 | 187 | 183 |
|  | IQR | 218 | 220 | 244 | 208 | 216 | 217 |
| Mild AD | Mean | 465 | 520 | 488 | 349 | 324 | 320 |
|  | SD | 3300 | 4186 | 3736 | 1870 | 1331 | 1581 |
|  | SE | 266 | 307 | 268 | 138 | 103 | 136 |
|  | Median | 156 | 164 | 168 | 161 | 150 | 141 |
|  | IQR | 198 | 185 | 204 | 148 | 123 | 137 |
| Moderate AD | Mean | 295 | 316 | 274 | 332 | 439 | 465 |
|  | SD | 593 | 781 | 568 | 999 | 2651 | 2877 |
|  | SE | 42 | 53 | 38 | 69 | 192 | 227 |
|  | Median | 129 | 150 | 143 | 125 | 143 | 139 |
|  | IQR | 163 | 149 | 145 | 187 | 154 | 143 |
| Moderately severe/severe AD | Mean | 382 | 370 | 340 | 315 | 285 | 233 |
|  | SD | 809 | 688 | 683 | 648 | 501 | 415 |
|  | SE | 55 | 44 | 44 | 44 | 37 | 34 |
|  | Median | 153 | 167 | 150 | 146 | 146 | 122 |
|  | IQR | 162 | 232 | 186 | 182 | 193 | 165 |
| **Public hospital stays, €** | | | | | | | |
| **Geriatric ward** | | | | | | | |
| Total | Mean | 234 | 356 | 627 | 532 | 763 | 735 |
|  | SD | 9830 | 10,184 | 11,754 | 15,931 | 12,687 | 15,238 |
|  | SE | 1766 | 1326 | 1164 | 1904 | 1384 | 1984 |
|  | Median | 10,784 | 9197 | 10,725 | 10,281 | 12,262 | 12,257 |
|  | IQR | 9694 | 8721 | 8941 | 7019 | 10,298 | 6824 |
| SCC | Mean | 58 | 265 | 313 | 261 | 357 | 256 |
|  | SD | 4356 | 11,709 | 7140 | 7580 | 9425 | 13,671 |
|  | SE | 2515 | 3703 | 1597 | 2102 | 2433 | 5581 |
|  | Median | 13,623 | 15,815 | 8428 | 10,254 | 9806 | 14,556 |
|  | IQR | 5085 | 16,544 | 5831 | 5796 | 6750 | 15,237 |
| MCI | Mean | 305 | 417 | 519 | 705 | 525 | 682 |
|  | SD | 8190 | 10,601 | 11,198 | 15,491 | 9324 | 14,344 |
|  | SE | 2469 | 2313 | 2011 | 3098 | 1944 | 3058 |
|  | Median | 19,882 | 9243 | 9176 | 11,951 | 8350 | 9894 |
|  | IQR | 11,759 | 10,452 | 8823 | 10,572 | 11,959 | 8118 |
| Mild AD | Mean | 485 | 307 | 965 | 237 | 811 | 1770 |
|  | SD | 16,055 | 10,078 | 16,988 | 5170 | 17,434 | 16,518 |
|  | SE | 7180 | 3359 | 4120 | 1723 | 5257 | 4768 |
|  | Median | 10,784 | 658 | 9006 | 3697 | 4903 | 22,353 |
|  | IQR | 18,996 | 8852 | 12,294 | 4496 | 10,722 | 14,991 |
| Moderate AD | Mean | 245 | 473 | 948 | 888 | 1387 | 540 |
|  | SD | 7863 | 10,942 | 11,645 | 30,601 | 10,771 | 6316 |
|  | SE | 3210 | 3460 | 3007 | 9677 | 2471 | 2233 |
|  | Median | 10,014 | 10,716 | 16,823 | 13,039 | 16,854 | 11,494 |
|  | IQR | 13,564 | 15,974 | 11,706 | 18,323 | 13,680 | 3069 |
| Moderately severe/severe AD | Mean | 297 | 364 | 1067 | 684 | 1741 | 1385 |
|  | SD | 10,593 | 5052 | 11,025 | 9592 | 16,403 | 19,838 |
|  | SE | 4325 | 1684 | 2529 | 2660 | 4101 | 5981 |
|  | Median | 7109 | 9197 | 12,257 | 9197 | 16,354 | 13,790 |
|  | IQR | 12,850 | 1335 | 6701 | 4946 | 7624 | 7627 |
| **Psychiatric ward** | | | | | | | |
| Total | Mean | 144 | 300 | 235 | 375 | 269 | 213 |
|  | SD | 14,689 | 30,722 | 24,810 | 47,664 | 21,152 | 25,166 |
|  | SE | 3672 | 5912 | 4962 | 9939 | 5130 | 6104 |
|  | Median | 12,180 | 11,637 | 9509 | 15,664 | 20,902 | 14,666 |
|  | IQR | 14,478 | 15,294 | 13,836 | 10,647 | 10,412 | 9847 |
| SCC | Mean | 127 | 152 | 86 | 114 | 62 | 116 |
|  | SD | 13,278 | 9362 | 9324 | 10,018 | 9682 | 8872 |
|  | SE | 5421 | 3121 | 3807 | 4480 | 5590 | 3968 |
|  | Median | 7045 | 5645 | 7548 | 12,466 | 9167 | 11,582 |
|  | IQR | 15,038 | 7818 | 6832 | 9717 | 12,805 | 12,975 |
| MCI | Mean | 59 | 376 | 462 | 363 | 376 | 195 |
|  | SD | 8491 | 30,680 | 32,146 | 41,453 | 27,889 | 8365 |
|  | SE | 6004 | 10,227 | 8591 | 15,668 | 11,385 | 3162 |
|  | Median | 16,694 | 11,637 | 8292 | 16,047 | 24,549 | 15,225 |
|  | IQR |  | 17,386 | 9205 | 11,997 | 33,974 | 10,538 |
| Mild AD | Mean | 155 | 1030 | 202 | 738 | 748 | 873 |
|  | SD | 9016 | 55,093 | 9659 | 19,781 | 19,673 | 51,393 |
|  | SE | 6375 | 24,639 | 6830 | 8076 | 9836 | 25,697 |
|  | Median | 13,921 | 27,093 | 21,292 | 19,326 | 23,149 | 11,020 |
|  | IQR |  | 16,318 |  | 9820 | 14,075 | 41,814 |
| Moderate AD | Mean | 24 | 65 | 207 | 47 | 22 | 123 |
|  | SD | 1527 | 3736 | 4607 | 6757 |  |  |
|  | SE | 1080 | 2157 | 3258 | 4778 |  |  |
|  | Median | 2689 | 3648 | 26,280 | 5326 | 4510 | 21,434 |
|  | IQR |  | 4676 |  |  |  |  |
| Moderately severe/severe AD | Mean | 502 | 111 | 101 | 1081 | 368 |  |
|  | SD | 19,056 |  |  | 114,518 | 13,687 |  |
|  | SE | 9528 |  |  | 66,117 | 7902 |  |
|  | Median | 22,820 | 27,934 | 26,064 | 27,681 | 19,233 |  |
|  | IQR | 19,818 |  |  | 139,119 | 17,437 |  |
| **Internal medicine ward** | | | | | | | |
| Total | Mean | 42 | 56 | 41 | 18 | 39 | 46 |
|  | SD | 12,906 | 14,347 | 30,385 | 6672 | 14,004 | 9253 |
|  | SE | 4878 | 5423 | 15,193 | 3852 | 6263 | 3778 |
|  | Median | 7568 | 13,510 | 7686 | 9197 | 7664 | 9196 |
|  | IQR | 8187 | 9696 | 23,676 | 8650 | 3659 | 14,213 |
| SCC | Mean | 86 | 52 | 118 | 25 | 109 | 73 |
|  | SD | 19,311 | 1062 | 40,051 | 2676 | 15,666 | 5247 |
|  | SE | 11,149 | 751 | 28,320 | 1892 | 7833 | 3710 |
|  | Median | 10,508 | 16,073 | 37,564 | 7304 | 8726 | 15,968 |
|  | IQR | 24,765 |  |  |  | 13,221 |  |
| MCI | Mean | 28 | 86 | 2 | 33 | 12 | 55 |
|  | SD | 6069 | 28,108 |  |  |  | 15,247 |
|  | SE | 4292 | 19,875 |  |  |  | 10,782 |
|  | Median | 7970 | 26,006 | 1086 | 18,386 | 6129 | 12,201 |
|  | IQR |  |  |  |  |  |  |
| Mild AD | Mean |  | 117 |  |  |  | 9 |
|  | SD |  | 4964 |  |  |  |  |
|  | SE |  | 2866 |  |  |  |  |
|  | Median |  | 6118 |  |  |  | 1359 |
|  | IQR |  | 6290 |  |  |  |  |
| Moderate AD | Mean |  |  |  |  |  |  |
|  | SD |  |  |  |  |  |  |
|  | SE |  |  |  |  |  |  |
|  | Median |  |  |  |  |  |  |
|  | IQR |  |  |  |  |  |  |
| Moderately severe/severe AD | Mean | 39 |  | 24 |  |  | 37 |
|  | SD | 4391 |  |  |  |  |  |
|  | SE | 3105 |  |  |  |  |  |
|  | Median | 4463 |  | 6129 |  |  | 6134 |
|  | IQR |  |  |  |  |  |  |
| **Surgery ward** | | | | | | | |
| Total | Mean | 152 | 285 | 225 | 214 | 229 | 158 |
|  | SD | 8168 | 17,449 | 13,638 | 17,855 | 9536 | 6272 |
|  | SE | 1444 | 2692 | 1948 | 3502 | 1713 | 1207 |
|  | Median | 6858 | 7350 | 3739 | 6562 | 9327 | 5607 |
|  | IQR | 4891 | 9755 | 3027 | 12,096 | 5965 | 7367 |
| SCC | Mean | 133 | 182 | 252 | 180 | 255 | 128 |
|  | SD | 13,804 | 8502 | 10,406 | 8966 | 7725 | 5137 |
|  | SE | 4881 | 2126 | 2219 | 2487 | 2443 | 1624 |
|  | Median | 4292 | 3884 | 3661 | 5596 | 12,999 | 4252 |
|  | IQR | 4947 | 5262 | 4453 | 6452 | 4376 | 2419 |
| MCI | Mean | 204 | 279 | 188 | 313 | 269 | 125 |
|  | SD | 5898 | 15,355 | 9448 | 27,594 | 11,672 | 7986 |
|  | SE | 1636 | 4630 | 2291 | 10,429 | 3369 | 3260 |
|  | Median | 7238 | 8319 | 3510 | 15,086 | 8352 | 8052 |
|  | IQR | 7646 | 11,417 | 3167 | 35,436 | 7634 | 11,353 |
| Mild AD | Mean | 102 | 445 | 119 | 84 | 264 | 325 |
|  | SD | 273 | 18,654 | 4002 | 9851 | 12,625 | 6566 |
|  | SE | 193 | 7615 | 1790 | 6965 | 6313 | 2680 |
|  | Median | 9151 | 10,373 | 3739 | 8067 | 7062 | 7522 |
|  | IQR |  | 10,717 | 1501 |  | 11,778 | 8511 |
| Moderate AD | Mean | 166 | 55 | 519 | 105 | 88 | 115 |
|  | SD | 7673 | 5626 | 29,627 |  | 4134 | 1712 |
|  | SE | 3431 | 3248 | 14,814 |  | 2387 | 1210 |
|  | Median | 4453 | 1115 | 31,806 | 24,106 | 7478 | 10,007 |
|  | IQR | 8527 | 6496 | 45,592 |  | 5269 |  |
| Moderately severe/severe AD | Mean | 99 | 638 | 51 | 278 | 178 | 213 |
|  | SD | 3161 | 32,288 |  | 18,680 | 1904 | 8054 |
|  | SE | 1581 | 13,182 |  | 10,785 | 1347 | 4650 |
|  | Median | 6712 | 13,048 | 13,082 | 17,495 | 17,342 | 11,934 |
|  | IQR | 3465 | 42,609 |  | 24,553 |  | 10,737 |
| **Others** | | | | | | | |
| Total | Mean | 718 | 1035 | 1294 | 1597 | 1400 | 1037 |
|  | SD | 11,365 | 11,564 | 10,770 | 15,801 | 14,495 | 13,776 |
|  | SE | 845 | 796 | 446 | 990 | 1000 | 1107 |
|  | Median | 2804 | 4973 | 1359 | 5459 | 5981 | 3656 |
|  | IQR | 3347 | 7224 | 873 | 7255 | 7641 | 7264 |
| SCC | Mean | 326 | 976 | 1363 | 1091 | 769 | 437 |
|  | SD | 7520 | 11,799 | 11,194 | 11,529 | 11,664 | 6456 |
|  | SE | 1290 | 1464 | 772 | 1340 | 1559 | 1076 |
|  | Median | 3267 | 3195 | 1358 | 4007 | 2982 | 2907 |
|  | IQR | 3040 | 7069 | 848 | 4685 | 4540 | 3504 |
| MCI | Mean | 929 | 1407 | 1403 | 1885 | 1386 | 1000 |
|  | SD | 12,099 | 14,644 | 11,237 | 14,132 | 12,028 | 11,189 |
|  | SE | 1457 | 1750 | 783 | 1442 | 1417 | 1482 |
|  | Median | 2643 | 7714 | 1359 | 6855 | 5655 | 3066 |
|  | IQR | 3819 | 8043 | 1328 | 5735 | 7560 | 4842 |
| Mild AD | Mean | 1046 | 846 | 1286 | 2019 | 2154 | 2140 |
|  | SD | 7296 | 5658 | 9249 | 19,771 | 21,539 | 25,188 |
|  | SE | 1310 | 1069 | 1349 | 3391 | 4397 | 5252 |
|  | Median | 2923 | 3650 | 1378 | 2888 | 5737 | 4597 |
|  | IQR | 4793 | 4309 | 1359 | 3685 | 12,804 | 11,656 |
| Moderate AD | Mean | 1102 | 485 | 1113 | 1960 | 1713 | 1064 |
|  | SD | 17,737 | 7160 | 6917 | 23,223 | 10,189 | 9942 |
|  | SE | 3547 | 1790 | 878 | 4389 | 1892 | 2411 |
|  | Median | 4597 | 3830 | 1359 | 7327 | 9051 | 5893 |
|  | IQR | 4780 | 7134 | 1373 | 8903 | 7372 | 16,734 |
| Moderately severe/severe AD | Mean | 559 | 959 | 1049 | 1434 | 2079 | 1673 |
|  | SD | 9565 | 7472 | 12,204 | 16,313 | 19,650 | 13,220 |
|  | SE | 2039 | 1321 | 1616 | 3401 | 3649 | 2818 |
|  | Median | 2731 | 4626 | 1359 | 8584 | 9847 | 8088 |
|  | IQR | 1552 | 5408 | 1375 | 11,593 | 12,246 | 10,554 |

AD, Alzheimer’s disease; IQR, interquartile range; MCI, mild cognitive impairment; SCC, subjective cognitive complaint; SD, standard deviation; SE, standard error of the mean.

|  |
| --- |
