# Supplementary material for: Economic and caregiver impact of Alzheimer’s disease across the disease spectrum: a cohort study
Source: Alzheimers Res Ther. 2022 Feb 12;14:34. doi: 10.1186/s13195-022-00969-x (PMC8841058; doi:10.1186/s13195-022-00969-x)
Supplement: Supplementary file 6 — Additional file 6: Table S5. Cost by Characteristics. [file 13195_2022_969_MOESM6_ESM.doc]

**Supplementary Table 5 Costs by age group, sex, education level, and treatment for comorbidities for each diagnosis**

|  | **Populations by age group (< 80 or ≥ 80 years old) and diagnosis** | | | | | | | | |
| --- | --- | --- | --- | --- | --- | --- | --- | --- | --- |
|  | | **Total  (n = 1998)** | **SCC (n = 640)** | **MCI  (n = 792)** | **Mild AD dementia (n = 121)** | **Moderate AD dementia (n = 211)** | **Moderately severe/severe AD dementia (n = 216)** | **P value** |  |
| < 80 years, n | | 941 | 427 | 292 | 69 | 68 | 85 | < .0001 |  |
| % | | 47.10 | 66.72 | 46.35 | 32.55 | 26.56 | 32.69 |  |
| ≥ 80 years, n | | 1057 | 213 | 338 | 143 | 188 | 175 |  |
| % | | 52.90 | 33.28 | 53.65 | 67.45 | 73.44 | 67.31 |  |

| **Costs** | | | | | | | | | | | | | | | |
| --- | --- | --- | --- | --- | --- | --- | --- | --- | --- | --- | --- | --- | --- | --- | --- |
|  |  | | **Second semester before** | | **First semester before** | | **First semester after** | | | **Second semester after** | | **Third semester after** | | **Fourth semester after** | |
| **By age group, €** | | | | | | | | | | | | | | | |
| < 80 years | SCC | | 3494 | | 3880 | | 4482 | | | 3418 | | 3657 | | 2418 | |
|  | MCI | | 3673 | | 4891 | | 4728 | | | 4860 | | 4469 | | 4139 | |
|  | Mild AD dementia | | 2622 | | 4464 | | 3509 | | | 4272 | | 6941 | | 7895 | |
|  | Moderate AD dementia | | 3385 | | 3604 | | 6452 | | | 6014 | | 5409 | | 6098 | |
|  | Moderately severe/severe AD dementia | | 3164 | | 4000 | | 3131 | | | 6066 | | 3924 | | 3940 | |
|  | P value | | .55 | | .28 | | .004 | | | .001 | | .01 | | < .001 | |
| ≥ 80 years | SCC | | 2395 | | 4861 | | 4770 | | | 4843 | | 4101 | | 3505 | |
|  | MCI | | 3055 | | 4796 | | 5002 | | | 6075 | | 4957 | | 4412 | |
|  | Mild AD dementia | | 4038 | | 5124 | | 6143 | | | 6576 | | 6954 | | 7523 | |
|  | Moderate AD dementia | | 2996 | | 3157 | | 5074 | | | 5654 | | 6292 | | 3799 | |
|  | Moderately severe/severe AD dementia | | 3565 | | 4944 | | 5851 | | | 6197 | | 8326 | | 6660 | |
|  | P value | | .02 | | .005 | | .27 | | | .27 | | < .001 | | < .001 | |
| Total | < 80 years | | 3450 | | 4229 | | 4509 | | | 4358 | | 4331 | | 3850 | |
|  | ≥ 80 years | | 3112 | | 4592 | | 5263 | | | 5836 | | 5797 | | 4863 | |
|  | P value | | .13 | | .21 | | .007 | | | < .001 | | < .001 | | .002 | |
| **By sex, €** | | | | | | | | | | | | | | | |
| Female | SCC | | 2117 | | 3096 | | 4162 | | | 4023 | | 3958 | | 2811 | |
|  | MCI | | 2785 | | 4379 | | 4124 | | | 5712 | | 4416 | | 4205 | |
|  | Mild AD dementia | | 2663 | | 3854 | | 4125 | | | 5597 | | 6197 | | 6840 | |
|  | Moderate AD dementia | | 3176 | | 3251 | | 5512 | | | 6070 | | 5931 | | 4306 | |
|  | Moderately severe/severe AD dementia | | 2979 | | 3676 | | 5183 | | | 5826 | | 5517 | | 5973 | |
|  | P value | | .01 | | .01 | | .03 | | | .004 | | .003 | | < .001 | |
| Male | SCC | | 4453 | | 5717 | | 5147 | | | 3736 | | 3620 | | 2835 | |
|  | MCI | | 4234 | | 5588 | | 6079 | | | 5179 | | 5263 | | 4422 | |
|  | Mild AD dementia | | 4965 | | 6628 | | 7139 | | | 6195 | | 8170 | | 8945 | |
|  | Moderate AD dementia | | 2875 | | 3327 | | 5264 | | | 4808 | | 6374 | | 4922 | |
|  | Moderately severe/severe AD dementia | | 4590 | | 7038 | | 4432 | | | 6992 | | 10,537 | | 4866 | |
|  | P value | | .24 | | .03 | | .12 | | | .003 | | < .001 | | < .001 | |
| Total | Female | | 2660 | | 3881 | | 4495 | | | 5280 | | 4856 | | 4343 | |
|  | Male | | 4303 | | 5692 | | 5611 | | | 4898 | | 5565 | | 4469 | |
|  | P value | | < .001 | | < .001 | | < .001 | | | .28 | | .06 | | .71 | |
| **By education level, €** | | | | | | | | | | | | | | | |
| < 12 years | | SCC | | 3207 | | 4447 | | 4785 | 4845 | | 4241 | | 3266 | |  |
|  | | MCI | | 3358 | | 5169 | | 4696 | 5636 | | 4824 | | 4279 | |  |
|  | | Mild AD dementia | | 3934 | | 5653 | | 5608 | 5952 | | 7046 | | 8713 | |  |
|  | | Moderate AD dementia | | 2939 | | 3233 | | 5728 | 6069 | | 5702 | | 4289 | |  |
|  | | Moderately severe/severe AD dementia | | 3520 | | 4440 | | 4929 | 6226 | | 6211 | | 6010 | |  |
|  | | P value | | .46 | | .001 | | .28 | .32 | | .003 | | < .001 | |  |
| ≥ 12 years | | SCC | | 2137 | | 3238 | | 3592 | 2577 | | 2654 | | 2251 | |  |
|  | | MCI | | 2562 | | 2837 | | 4158 | 4928 | | 3737 | | 3909 | |  |
|  | | Mild AD dementia | | 2632 | | 3246 | | 4565 | 5509 | | 6719 | | 4938 | |  |
|  | | Moderate AD dementia | | 3849 | | 3462 | | 4044 | 4198 | | 7664 | | 5351 | |  |
|  | | Moderately severe/severe AD dementia | | 2800 | | 6345 | | 5296 | 5680 | | 11,497 | | 3007 | |  |
|  | | P value | | .11 | | .10 | | .36 | < .001 | | < .001 | | < .001 | |  |
| Total | | < 12 years | | 3350 | | 4635 | | 5023 | 5664 | | 5302 | | 4826 | |  |
|  | | ≥ 12 years | | 2443 | | 3299 | | 3952 | 3755 | | 4164 | | 3242 | |  |
|  | | P value | | < .001 | | < .001 | | < .001 | < .001 | | .002 | | < .001 | |  |
| **By treatment for comorbidities, €** | | | | | | | | | | | | | | | |
| **Hypertension** | | | | | | | | | | | | | | | |
| No hypertension | SCC | | 2198 | | 2609 | | 3916 | | | 1842 | | 2114 | | 1225 | |
|  | MCI | | 2122 | | 2671 | | 4061 | | | 3857 | | 4532 | | 4468 | |
|  | Mild AD dementia | | 3238 | | 5740 | | 5487 | | | 4160 | | 4070 | | 11,195 | |
|  | Moderate AD dementia | | 2478 | | 2342 | | 3637 | | | 5256 | | 6048 | | 3372 | |
|  | Moderately severe/severe AD dementia | | 3301 | | 4132 | | 6093 | | | 7080 | | 9800 | | 3900 | |
|  | P value | | .13 | | < .001 | | .048 | | | < .001 | | < .001 | | < .001 | |
| Hypertension | SCC | | 3781 | | 5388 | | 5063 | | | 5385 | | 4996 | | 3939 | |
|  | MCI | | 3785 | | 5661 | | 5201 | | | 6146 | | 4814 | | 4212 | |
|  | Mild AD dementia | | 3681 | | 4556 | | 5190 | | | 6521 | | 8256 | | 5827 | |
|  | Moderate AD dementia | | 3507 | | 3868 | | 6635 | | | 6036 | | 6037 | | 5066 | |
|  | Moderately severe/severe AD dementia | | 3490 | | 4867 | | 4448 | | | 5784 | | 5706 | | 6360 | |
|  | P value | | .95 | | .04 | | .045 | | | .60 | | .002 | | .002 | |
| Total | No hypertension | | 2431 | | 3055 | | 4317 | | | 3625 | | 4321 | | 3768 | |
|  | Hypertension | | 3700 | | 5141 | | 5227 | | | 5911 | | 5528 | | 4706 | |
|  | P value | | < .001 | | < .001 | | .001 | | | < .001 | | < .001 | | .004 | |
| **Diabetes mellitus** | | | | | | | | | | | | | | | |
| No diabetes mellitus | SCC | | 2586 | | 3669 | | 4073 | | | 3367 | | 3504 | | 2823 | |
|  | MCI | | 2751 | | 4400 | | 4120 | | | 4911 | | 4411 | | 3866 | |
|  | Mild AD dementia | | 3588 | | 4773 | | 4545 | | | 5837 | | 6324 | | 7314 | |
|  | Moderate AD dementia | | 2930 | | 2985 | | 4689 | | | 5447 | | 6121 | | 4299 | |
|  | Moderately severe/severe AD dementia | | 3223 | | 4528 | | 5008 | | | 6858 | | 6777 | | 5508 | |
|  | P value | | .12 | | .003 | | .23 | | | < .001 | | < .001 | | < .001 | |
| Diabetes mellitus | SCC | | 5563 | | 6739 | | 6987 | | | 6366 | | 5287 | | 2810 | |
|  | MCI | | 6004 | | 6837 | | 8387 | | | 8339 | | 6373 | | 6360 | |
|  | Mild AD dementia | | 3471 | | 5324 | | 7716 | | | 5781 | | 8841 | | 8639 | |
|  | Moderate AD dementia | | 4006 | | 4992 | | 10,010 | | | 7487 | | 5617 | | 5272 | |
|  | Moderately severe/severe AD dementia | | 4214 | | 5054 | | 4816 | | | 3286 | | 7299 | | 6385 | |
|  | P value | | .09 | | .39 | | .02 | | | .001 | | .19 | | < .001 | |
| Total | No diabetes | | 2857 | | 4031 | | 4336 | | | 4822 | | 4820 | | 4133 | |
|  | Diabetes | | 5056 | | 6155 | | 7502 | | | 6569 | | 6445 | | 5483 | |
|  | P value | | < .001 | | < .001 | | < .001 | | | < .001 | | .001 | | .003 | |
| **Hypercholesterolemia** | | | | | | | | | | | | | | | |
| No hypercholesterolemia | SCC | | 2373 | | 3437 | | 3814 | | | 3318 | | 3113 | | 2501 | |
|  | MCI | | 3198 | | 5032 | | 4617 | | | 5618 | | 4577 | | 4270 | |
|  | Mild AD dementia | | 3739 | | 4732 | | 4916 | | | 6910 | | 5880 | | 7830 | |
|  | Moderate AD dementia | | 3337 | | 3491 | | 5359 | | | 5902 | | 5836 | | 4334 | |
|  | Moderately severe/severe AD dementia | | 3483 | | 4268 | | 4869 | | | 7104 | | 6802 | | 5484 | |
|  | P value | | .004 | | .003 | | .03 | | | < .001 | | < .001 | | < .001 | |
| Hypercholesterolemia | SCC | | 4615 | | 5763 | | 6121 | | | 5057 | | 5194 | | 3458 | |
|  | MCI | | 3532 | | 4591 | | 5210 | | | 5369 | | 4929 | | 4305 | |
|  | Mild AD dementia | | 3267 | | 5204 | | 5906 | | | 3998 | | 8817 | | 7344 | |
|  | Moderate AD dementia | | 2620 | | 2820 | | 5620 | | | 5445 | | 6438 | | 4717 | |
|  | Moderately severe/severe AD dementia | | 3339 | | 5361 | | 5169 | | | 4326 | | 7052 | | 6104 | |
|  | P value | | .02 | | .002 | | .62 | | | .37 | | .01 | | .001 | |
| Total | No hypercholesterolemia | | 3022 | | 4146 | | 4514 | | | 5213 | | 4693 | | 4237 | |
|  | Hypercholesterolemia | | 3692 | | 4892 | | 5588 | | | 5016 | | 5826 | | 4641 | |
|  | P value | | .004 | | .02 | | < .001 | | | .58 | | .003 | | .23 | |
| **Depression** | | | | | | | | | | | | | | | |
| No depression | SCC | | 3634 | | 4432 | | 4329 | | | 3355 | | 3036 | | 2092 | |
|  | MCI | | 2796 | | 5109 | | 4614 | | | 5017 | | 4546 | | 4258 | |
|  | Mild AD dementia | | 3445 | | 3814 | | 4827 | | | 4587 | | 5617 | | 7014 | |
|  | Moderate AD dementia | | 3207 | | 3051 | | 4524 | | | 3943 | | 5173 | | 3500 | |
|  | Moderately severe/severe AD dementia | | 3425 | | 5257 | | 5171 | | | 3623 | | 6879 | | 5439 | |
|  | P value | | .24 | | .01 | | .79 | | | .008 | | < .001 | | < .001 | |
| Depression | SCC | | 2331 | | 3878 | | 4949 | | | 4691 | | 4919 | | 3786 | |
|  | MCI | | 4031 | | 4488 | | 5226 | | | 6132 | | 4968 | | 4321 | |
|  | Mild AD dementia | | 3664 | | 5926 | | 5716 | | | 6950 | | 8179 | | 8233 | |
|  | Moderate AD dementia | | 2983 | | 3499 | | 6437 | | | 7573 | | 6877 | | 5401 | |
|  | Moderately severe/severe AD dementia | | 3441 | | 4141 | | 4812 | | | 7928 | | 6884 | | 5861 | |
|  | P value | | < .001 | | .03 | | .23 | | | .001 | | .002 | | < .001 | |
| Total | No depression | | 3271 | | 4524 | | 4582 | | | 4117 | | 4414 | | 3810 | |
|  | Depression | | 3269 | | 4299 | | 5303 | | | 6313 | | 5909 | | 5030 | |
|  | P value | | .99 | | .44 | | .01 | | | < .001 | | < .001 | | < .001 | |
| **Anxiety** | | | | | | | | | | | | | | | |
| No anxiety | SCC | | 2876 | | 3642 | | 4390 | | | 3166 | | 3434 | | 2362 | |
|  | MCI | | 2763 | | 4073 | | 3876 | | | 5103 | | 3988 | | 4112 | |
|  | Mild AD dementia | | 3153 | | 3275 | | 4367 | | | 4247 | | 4893 | | 7839 | |
|  | Moderate AD dementia | | 2950 | | 3389 | | 5509 | | | 6292 | | 5367 | | 4131 | |
|  | Moderately severe/severe AD dementia | | 3378 | | 4316 | | 3529 | | | 3385 | | 6958 | | 4666 | |
|  | P value | | .67 | | .28 | | .01 | | | < .001 | | < .001 | | < .001 | |
| Anxiety | SCC | | 3683 | | 5579 | | 5028 | | | 5600 | | 4722 | | 3941 | |
|  | MCI | | 4457 | | 6320 | | 6861 | | | 6286 | | 6185 | | 4628 | |
|  | Mild AD dementia | | 4410 | | 8531 | | 7431 | | | 9493 | | 12,010 | | 7173 | |
|  | Moderate AD dementia | | 3358 | | 3049 | | 5309 | | | 4759 | | 7315 | | 5045 | |
|  | Moderately severe/severe AD dementia | | 3519 | | 5116 | | 7115 | | | 9959 | | 6772 | | 7102 | |
|  | P value | | .42 | | < .001 | | .03 | | | .001 | | .001 | | .01 | |
| Total | No anxiety | | 2936 | | 3785 | | 4270 | | | 4307 | | 4401 | | 4028 | |
|  | Anxiety | | 3938 | | 5712 | | 6227 | | | 6779 | | 6563 | | 5117 | |
|  | P value | | < .001 | | < .001 | | < .001 | | | < .001 | | < .001 | | .002 | |

AD, Alzheimer’s disease; IQR, interquartile range; MCI, mild cognitive impairment; SCC, subjective cognitive complaint; SD, standard deviation; SE, standard error of the mean.
