# Supplementary material for: Economic and caregiver impact of Alzheimer’s disease across the disease spectrum: a cohort study
Source: Alzheimers Res Ther. 2022 Feb 12;14:34. doi: 10.1186/s13195-022-00969-x (PMC8841058; doi:10.1186/s13195-022-00969-x)
Supplement: Supplementary file 7 — Additional file 7: Table S6. Sensitivity. [file 13195_2022_969_MOESM7_ESM.doc]

**Supplementary Table 6: Sensitivity analysis**

|  | |  | | **Second semester before (n = 1714)** | | **First semester before (n = 1844)** | | **First semester after (n = 1947)** | | **Second semester after (n = 1735)** | | **Third semester after (n = 1541)** | | **Fourth semester after (n = 1340)** |
| --- | --- | --- | --- | --- | --- | --- | --- | --- | --- | --- | --- | --- | --- | --- |
| **Mean total cost per semester for each diagnosis after exclusion of costs ≥ 3 SD of the average total cost, €** | | | | | | | | | | | | | | |
| SCC | | Mean | | 1786 | | 2552 | | 3582 | | 3140 | | 2862 | | 2286 |
|  | | SE | | 128 | | 206 | | 229 | | 257 | | 238 | | 188 |
|  | | Median | | 736 | | 799 | | 1661 | | 814 | | 861 | | 848 |
|  | | SD | | 3027 | | 4992 | | 5701 | | 6094 | | 5301 | | 3899 |
|  | | IQR | | 817 | | 984 | | 1697 | | 1216 | | 1363 | | 1122 |
| MCI | | Mean | | 1972 | | 2774 | | 3391 | | 3759 | | 3371 | | 3246 |
|  | | SE | | 138 | | 206 | | 190 | | 246 | | 236 | | 236 |
|  | | Median | | 836 | | 1053 | | 1652 | | 1361 | | 1348 | | 1331 |
|  | | SD | | 3207 | | 4946 | | 4678 | | 5747 | | 5251 | | 4930 |
|  | | IQR | | 915 | | 963 | | 1736 | | 2037 | | 1956 | | 2047 |
| Mild AD dementia | | Mean | | 2189 | | 3267 | | 3506 | | 3660 | | 4314 | | 3232 |
|  | | SE | | 277 | | 375 | | 282 | | 304 | | 452 | | 336 |
|  | | Median | | 824 | | 1307 | | 2179 | | 2352 | | 2604 | | 2329 |
|  | | SD | | 3638 | | 5282 | | 4017 | | 4148 | | 5812 | | 4009 |
|  | | IQR | | 1019 | | 1542 | | 1984 | | 2538 | | 2467 | | 1864 |
| Moderate AD dementia | | Mean | | 2203 | | 3003 | | 4120 | | 3688 | | 4210 | | 3525 |
|  | | SE | | 230 | | 315 | | 386 | | 331 | | 444 | | 339 |
|  | | Median | | 772 | | 1077 | | 1938 | | 2157 | | 2059 | | 1945 |
|  | | SD | | 3410 | | 4840 | | 6063 | | 4925 | | 6243 | | 4426 |
|  | | IQR | | 1280 | | 1649 | | 2604 | | 2666 | | 2381 | | 2143 |
| Moderately severe/severe AD dementia | | Mean | | 2237 | | 3343 | | 3932 | | 3651 | | 4463 | | 3850 |
|  | | SE | | 209 | | 317 | | 328 | | 360 | | 460 | | 405 |
|  | | Median | | 820 | | 1151 | | 2091 | | 1948 | | 2110 | | 1809 |
|  | | SD | | 3117 | | 4955 | | 5222 | | 5347 | | 6296 | | 5140 |
|  | | IQR | | 1305 | | 2184 | | 3023 | | 2929 | | 3540 | | 3148 |
| P value* | |  | | .071 | | .025 | | .163 | | .159 | | < .001 | | < .001 |
| P value^†^ | |  | | .014 | | .016 | | .035 | | .848 | | .017 | | < .001 |
| **Mean total cost per semester by domain, €** | | | | | | | | | | | | | | |
| Ambulatory medicine (including transportation) | Mean | | 442 | | 643 | | 693 | | 546 | | 528 | | 422 | |
|  | SE | | 21 | | 43 | | 41 | | 34 | | 36 | | 17 | |
|  | Median | | 278 | | 332 | | 377 | | 261 | | 262 | | 242 | |
|  | SD | | 867 | | 1844 | | 1774 | | 1387 | | 1417 | | 620 | |
|  | IQR | | 268 | | 291 | | 324 | | 281 | | 257 | | 239 | |
| Paramedical medicine | Mean | | 514 | | 689 | | 954 | | 1065 | | 1149 | | 1144 | |
|  | SE | | 52 | | 49 | | 55 | | 56 | | 59 | | 63 | |
|  | Median | | 167 | | 232 | | 438 | | 623 | | 709 | | 737 | |
|  | SD | | 1684 | | 1740 | | 2042 | | 1971 | | 1997 | | 1976 | |
|  | IQR | | 408 | | 546 | | 953 | | 1238 | | 1335 | | 1351 | |
| Pharmaceutical treatment | Mean | | 422 | | 427 | | 422 | | 428 | | 395 | | 356 | |
|  | SE | | 24 | | 23 | | 19 | | 21 | | 21 | | 16 | |
|  | Median | | 207 | | 213 | | 248 | | 249 | | 245 | | 251 | |
|  | SD | | 944 | | 940 | | 786 | | 839 | | 785 | | 556 | |
|  | IQR | | 251 | | 254 | | 296 | | 265 | | 235 | | 253 | |
| Stays in public hospital | Mean | | 500 | | 911 | | 1339 | | 1269 | | 1277 | | 969 | |
|  | SE | | 330 | | 452 | | 229 | | 454 | | 547 | | 528 | |
|  | Median | | 2731 | | 4597 | | 1366 | | 5742 | | 6129 | | 4766 | |
|  | SD | | 4530 | | 6865 | | 5719 | | 7466 | | 8132 | | 6970 | |
|  | IQR | | 3158 | | 6895 | | 1361 | | 7278 | | 8109 | | 7108 | |
| Stays in private hospital | Mean | | 120 | | 191 | | 197 | | 217 | | 199 | | 153 | |
|  | SE | | 156 | | 267 | | 399 | | 437 | | 362 | | 423 | |
|  | Median | | 967 | | 868 | | 988 | | 1117 | | 1323 | | 1080 | |
|  | SD | | 1763 | | 3359 | | 4623 | | 5040 | | 4003 | | 3904 | |
|  | IQR | | 764 | | 1111 | | 1249 | | 1077 | | 1047 | | 956 | |

* GLM comparing mean total costs between diagnosis groups.

^†^ GLM adjusted for age, sex, and education level

AD, Alzheimer's disease; GLM, general linear model; IQR, interquartile range; MCI, mild cognitive impairment; SCC, subjective cognitive complaint; SD, standard deviation; SE, standard error of the mean.
