# Supplementary material for: Economic and caregiver impact of Alzheimer’s disease across the disease spectrum: a cohort study
Source: Alzheimers Res Ther. 2022 Feb 12;14:34. doi: 10.1186/s13195-022-00969-x (PMC8841058; doi:10.1186/s13195-022-00969-x)
Supplement: Supplementary file 8 — Additional file 8: Table S7. Characteristics of patients. [file 13195_2022_969_MOESM8_ESM.doc]

**Supplementary Table 7: Characteristics of patients with costs ≥3 SD of the average total cost for the first semester after the first memory clinic visit**

|  | < 3 SD | ≥ 3 SD | P value |
| --- | --- | --- | --- |
|  | (n = 1947) | (n = 51) |  |
| Age |  |  |  |
| ≥ 80 years, n (%) | 1027 (52.7) | 30 (58.8) | .391 |
| Sex |  |  |  |
| Female, n (%) | 1230 (63.2) | 29 (56.9) | .357 |
| Male, n (%) | 717 (36.8) | 22 (43.1) |  |
| Education level |  |  |  |
| ≥ 12 years, n (%) | 600 (30.8) | 10 (19.6) | .085 |
| < 12 years, n (%) | 1278 (65.6) | 37 (72.5.2) |  |
| Unknown, n (%) | 69 (3.5) | 4 (7.8) |  |
| MMSE (out of 30), mean (SD) | 22.1 (6.2) | 21.46 (5.49) | .466 |
| IADL (out of 8, mean (SD) | 5.09 (2.4) | 3.77 (2.33) | < .0001 |
| NPI (out of 144, mean (SD) | 18.47 (16.09) | 19.34 (17.23) | .761 |
|  | *n = 1364* | *n = 32* |  |
| Receiving treatment for comorbidities n (%) |  |  |  |
| Hypertension | 1263 (64.9) | 31 (60.8) | .547 |
| Diabetes mellitus | 344 (17.7) | 16 (31.4) | .012 |
| Hypercholesterolemia | 710 (36.5) | 21 (41.2) | .491 |
| Depression | 881 (45.2) | 21 (41.2) | .564 |
| Anxiety | 625 (32.1) | 25 (49) | .011 |

MMSE, Mini-Mental State Exam; IADL, Instrumental Activities of Daily Living; NPI, Neuropsychiatric Inventory
