# Supplementary material for: Economic and caregiver impact of Alzheimer’s disease across the disease spectrum: a cohort study
Source: Alzheimers Res Ther. 2022 Feb 12;14:34. doi: 10.1186/s13195-022-00969-x (PMC8841058; doi:10.1186/s13195-022-00969-x)
Supplement: Supplementary file 9 — Additional file 9: Table S8. Other Studies. [file 13195_2022_969_MOESM9_ESM.doc]

**Supplementary Table 8:** Comparison of studies in Europe presenting direct medical costs according to AD disease spectrum (and transportation when available)

|  |  |  |  |  |  | **AD dementia** | | | |
| --- | --- | --- | --- | --- | --- | --- | --- | --- | --- |
| **Author - year of publication** | **Localization of the study** | **N** | **Costs characteristics** | **SCC** | **MCI** | **Mild AD** | **Moderate AD** | | **Moderately/severe AD** |
| Bruno et al. – 2018 [34] | Italy - GERAS II study | 198 | RUD questionnaire* - Health care costs included patients's AD medications, antipsychotics/hypnotic medication and medications for co-morbodities, nights in hospital, emergency room and outpatients visits (costs in Euro 2013 value) | NA | NA | 2 520 € / year (210 € / month) | 1 560 € / year  (130 € / month) | | 1 272 € / year  (106 € / month) |
| Gervès et al. – 2014 [36] | France | 57 | RUD questionnaire* - Health care costs included medical care, AD-related drug, hospital stays, physicians and nursing services. (Costs in euros, reference year: NA, data collected between 2009 and 2010) | NA | NA | Mild (MMSE>19): 4 920€ / year  (410 € / month) | Moderate to severe (MMSE <=19):  7 464€ / year  (622 € / month) | | |
| Lenox-Smith et al. -2016 [6] | UK - GERAS study | 526 | RUD questionnaire* - Health care costs included patients's AD medications, antipsychotics/hypnotic medication and medications for co-morbodities, nights in hospital, emergency room and outpatients visits (costs in Euro 2010 value) | NA | NA | 4 920€ / year | 2 436€ / year  (3 130£ /18mois ≈ 3 654€) | 2 384€ / year  (3 055£ /18mois ≈ 3 576€) | |
| Luppa et al. – 2008 [33] | Germany | 452 | Health care costs included medical costs (outpatient services, non-physician provider, medical supply/dentures, inpatient care), pharmaceutical, transportation (non-medical cost) (costs in Euro 2004/2005 value) . | Cognitively normal (no information available on complaint)  3 414 € | 4 100 € | NA | NA | NA | |
| Olazaran et al. -2017 [5] | Spain - GERAS II study | 380 | RUD questionnaire* - Health care costs included medications, nights in hospital, emergency room, outpatients visits, costs of neuropsychological assessments (costs in Euro 2013 value) | NA | NA | 3 264 € / year (272€ / month) | 2 832 € / year(236€ / month) | 4 152€ / year(346€ / month) | |
| Rapp et al. – 2018 [37] | France - GERAS study | 419 | RUD questionnaire* - Health care costs included patients's AD medications, antipsychotics/hypnotic medication and medications for co-morbodities, nights in hospital, emergency room and outpatients visits (costs in Euro 2010 value) | NA | NA | 3 419 € / year  (5 129€ / 18 months) | 4 737 € / year  (7 106€ / 18 months) | 5 412 € / year  (8 118€ / 18 months) | |
| Rapp et al. – 2012 [35] | France - PLASA study | 815 | RUD questionnaire* -Medical costs included costs associated with inpatient and outpatient hospital visits, physician visits, speech therapist visits, physical therapist visits, nurse visits, and medication use. (Costs in euros, reference year: NA, data collected between 2003 and 2005) | NA | NA | Mild (MMSE>20): 405 € / year | Modarate to severe AD (MMSE<=20): 365 € / year | | |
| Rigaud et al. 2003 [27] | France | 50 | Questionnaire completed by the caregiver - Medical consumption including medical consultations, physiotherapy,speech therapy, occupational therapy, memory stimulation, visiting nurses during the past month,and hospitalizations during the past 6 months. (costs in Euro 1996 value) . In this table, costs of paid and unpaid informal assistance were not reported | Non-AD sample: 2592€ / year  (216€ / month) | | Patients with MMSE >21 3552€ / year (296€ / month) | Patients with MMSE between 16 and 20 4512€ / year  (376€ / month) | Patients with MMSE between 11-15: 5184€ / year (432€ / month)  Patients with MMSE <=10: 6276€ / year (523€ / month) | |
| Wimo et al. – 2013 [21] | France - Germany - UK - GERAS study | Total: 1497 (France: 419, Germany: 552, UK: 526) | RUD questionnaire* - Health care costs included patients's AD medications, antipsychotics/hypnotic medication and medications for co-morbodities, nights in hospital, emergency room and outpatients visits (costs in Euro 2010 value) | NA | NA | In France :  3 636 € / year (303 € / month) In Germany : 5 292 € / year (441 € / month) In UK :  2 016 € / year (168 € / month) | In France :  5 004 € / year (417 € / month) In Germany : 7 140 € / year (595 € / month) In UK :  2 556 € / year (213 € / month) | In France :  5 952 € / year (496 € / month)  In Germany :  8 712 € / year (726 € / month) In UK :  2 628 € / year (219 € / month) | |
| * RUD Ressource Utilization in Dementia NA Not available | | |  |  |  |  |  |  | |
| Only the healthcare medical costs (and transportation when available) were considered in this table | | | | | | |  |  | |
