# Supplementary material for: Economic and caregiver impact of Alzheimer’s disease across the disease spectrum: a cohort study
Source: Alzheimers Res Ther. 2022 Feb 12;14:34. doi: 10.1186/s13195-022-00969-x (PMC8841058; doi:10.1186/s13195-022-00969-x)
Supplement: Supplementary file 10 — Additional file 10: Table S9. Events. [file 13195_2022_969_MOESM10_ESM.doc]

**Supplementary Table 9: Deaths and nursing-home admissions for each diagnosis**

|  |  | Third semester | Fourth semester | Total,  2014-2019 | Delay before event (mean ± SD), in months*^,†^ |
| --- | --- | --- | --- | --- | --- |
| **Deaths per semester following the first memory clinic visit for each diagnosis** | | | | | |
| **SCC**, n | | 510 | 437 | 640 |  |
| Deaths, n | | 2 | 0 | 42 | 49.10 ± 23.60 |
| % | | 0.39 | 0 | 6.56 |  |
| **MCI**, n | | 509 | 446 | 630 |  |
| Deaths, n | | 6 | 10 | 103 | 45.10 ± 20.86 |
| % | | 1.18 | 2.24 | 16.35 |  |
| **Mild AD** **dementia**, n | | 173 | 153 | 212 |  |
| Deaths, n | | 3 | 4 | 56 | 51.52 ± 22.35 |
| % | | 1.73 | 2.61 | 26.42 |  |
| **Moderate AD dementia**, n | | 208 | 174 | 256 |  |
| Deaths, n | | 5 | 7 | 61 | 42.61 ± 21.02 |
| % | | 2.40 | 4.02 | 23.83 |  |
| **Moderately severe/severe AD dementia**, n | | 195 | 166 | 260 |  |
| Deaths, n | | 6 | 10 | 71 | 39.59 ± 19.19 |
| % | | 3.08 | 6.02 | 27.31 |  |
| **Total**, n | | 1595 | 1376 | 1998 |  |
| Deaths, n | | 22 | 31 | 333 | 45.05 ± 21.43 |
| % | | 1.38 | 2.25 | 16.67 |  |
| **Nursing home admissions per semester following the first memory clinic visit for each diagnosis** | | | | | |
| **SCC**, n | | 510 | 437 | 640 |  |
| Nursing home admissions, n | | 10 | 6 | 77 | 42.27 ± 21.89 |
| % | | 1.96 | 1.37 | 12.03 |  |
| **MCI**, n | | 509 | 446 | 630 |  |
| Nursing home admissions, n | | 23 | 24 | 146 | 37.13 ± 19.49 |
| % | | 4.52 | 5.38 | 23.17 |  |
| **Mild AD** **dementia**, n | | 173 | 153 | 212 |  |
| Nursing home admissions, n | | 18 | 17 | 85 | 35.55 ± 19.95 |
| % | | 10.40 | 11.11 | 40.09 |  |
| **Moderate AD dementia**, n | | 208 | 174 | 256 |  |
| Nursing home admissions, n | | 22 | 13 | 89 | 31.55 ± 17.83 |
| % | | 10.58 | 7.47 | 34.77 |  |
| **Moderately severe/severe AD dementia** - n | | 195 | 166 | 260 |  |
| Nursing home admissions, n | | 21 | 18 | 78 | 25.19 ± 10.66 |
| % | | 10.77 | 10.84 | 30.00 |  |
| **Total**, n | | 1595 | 1376 | 1998 |  |
| Nursing home admissions, n | | 94 | 78 | 333 | 34.68 ± 19.21 |
| % | | 5.89 | 5.67 | 16.67 |  |

*For patients attending a memory visit between 2014 and 2019.

^†^ The occurrence of deaths between 2014 and 2019.

AD, Alzheimer’s disease; IQR, interquartile range; MCI, mild cognitive impairment; SCC, subjective cognitive complaint; SD, standard deviation; SE, standard error of the mean.
